# Association of Metal Exposure with Novel Immunoinflammatory Indicators

**DOI:** 10.3390/toxics12050316

**Published:** 2024-04-26

**Authors:** Lingxiao Zhao, Xieyi Chen, Zhongwen Chen, Cantao Yang, Qiang Huang, Shuqun Cheng

**Affiliations:** 1 Department of Occupational and Environmental Health, School of Public Health, Chongqing Medical University, Yixueyuan Road, Yuzhong District, Chongqing 400016, China; zlxiao@stu.cqmu.edu.cn (L.Z.); 2023121639@stu.cqmu.edu.cn (X.C.); 2021120755@stu.cqmu.edu.cn (Z.C.); 2 Yubei District Center for Disease Control and Prevention, Chongqing 401120, China; 2020121518@stu.cqmu.edu.cn; 3 Chongqing Center for Disease Control and Prevention, Chongqing 400707, China; 18983832727@163.com

**Keywords:** metals, inflammation, national health and nutrition examination survey, gender, inflammatory index

## Abstract

**Objective:** We aimed to investigate the relationship between metal exposure and novel immunoinflammatory indicators. **Methods:** Data on adults participating in the National Health and Nutrition Examination Survey (NHANES) from 2009 to 2018 were analyzed. Various statistical models were employed to assess the association between metal exposure and novel immune-inflammation-related indicators. Additionally, the impact of metal exposure on inflammation in different gender populations was explored. **Results:** This study included 4482 participants, of whom 51.1% were male. Significant correlations were observed among various metals. Both elastic net (ENET) and linear regression models revealed robust associations between cadmium (Cd), cobalt (Co), arsenic (As), mercury (Hg), and immunoinflammatory indicators. Weighted quantile sum (WQS) and Quantile g-computation (Q-gcomp) models demonstrated strong associations between barium (Ba), Co, and Hg and immunoinflammatory indicators. Bayesian kernel machine regression (BKMR) analysis indicated an overall positive correlation between in vivo urinary metal levels and systemic inflammation response index (SIRI) and aggregate index of systemic inflammation (AISI). Furthermore, Co, As, and Hg emerged as key metals contributing to changes in novel immunoinflammatory indicators. **Conclusions:** Metals exhibit associations with emerging immunoinflammatory indicators, and concurrent exposure to mixed metals may exacerbate the inflammatory response. Furthermore, this relationship varies across gender populations.

## 1. Introduction

Metals are naturally occurring elements that are integral to numerous environmental and biological processes [1]. However, human activities, including industrial and agricultural practices, contribute to the release and migration of these metals, leading to environmental pollution and adverse impacts on ecosystems and human health [2]. Environmental problems caused by metals and their mixtures pose a significant global public health concern [3]. Compared with single-metal pollutants, compound pollutants (coexistence of multiple metals) are more common. Metal composite pollutants exhibit characteristics of universality and complexity, with uncertain ecological effects stemming from addition, antagonism, and synergism [4]. Human exposure to metal contaminants occurs through various routes, including the ingestion of contaminated water and food, inhalation of polluted air, and dermal contact [5]. Elevated levels of specific metals have been associated with various health issues. For instance, copper exposure has been associated with an increased risk of nonalcoholic fatty liver disease (NAFLD) [6], while heavy metals such as iron, mercury, manganese, cuprom, and lead have been implicated in the development of Parkinson’s disease [7]. Additionally, cadmium exposure has been linked to chronic kidney disease [8].

Immunoinflammatory indicators, including lymphocytes, neutrophils, and albumin, serve as hematologic indicators reflecting inflammatory or nutritional immune status [9]. These indicators are valuable for early disease detection and prognosis assessment. Previous research suggests that increased metals levels, such as Pb, Cd, Hg, and As, upregulate interleukin-6 (IL-6) expression, potentially leading to decreased lymphocyte levels [10]. Similarly, elevated Ni levels in patients with lung cancer correlate with increased IL-6 levels. However, single indicators like lymphocytes and interleukins are susceptible to confounding factors and may not provide a comprehensive assessment of the body’s inflammatory and immunotrophic status [11]. Novel inflammation indicators, such as the aggregate index of systemic inflammation (AISI), system inflammation response index (SIRI), and the Hemoglobin, Albumin, Lymphocyte, and Platelet (HALP) score, offer more accurate evaluations than single indicators by [12,13,14] integrating and analyzing various inflammatory cell levels, providing a holistic view of the body’s immune inflammatory status. The HALP score offers a comprehensive assessment of immuno-nutritional function with higher stability compared to traditional indicators. For instance, a study demonstrated that the HALP score can quickly assess the nutritional level in patients with bowel cancer [15]. SIRI dynamically changes with immune inflammatory responses, reflecting cancer progression and treatment efficacy [16], while AISI offers a comprehensive assessment of systemic inflammatory status through whole blood cells, providing a more holistic view compared to traditional indicators [17].

Emerging evidence indicates that metals can trigger Alzheimer’s disease through inflammatory responses [18,19], disrupting immune homeostasis and inducing inflammation [10,20,21]. Therefore, early monitoring of immune and inflammatory indicators aids in disease detection and prognosis prediction in metal-induced disorders. Novel indicators such as the HALP score, which assesses nutritional immunity and the inflammatory response [22], offer improved prognostic accuracy compared to indicators such as C-reactive protein. However, only a few studies have explored the relationship between metals and these novel inflammatory metrics, with existing research predominately focused on traditional inflammatory metrics. Therefore, this study aims to analyze the association between metal exposure and novel indicators, such as HALP scores, to enhance the prediction of adverse health outcomes stemming from metal exposure.

## 2. Materials and Methods

### 2.1. Study Design and Participant

**Figure 1:**
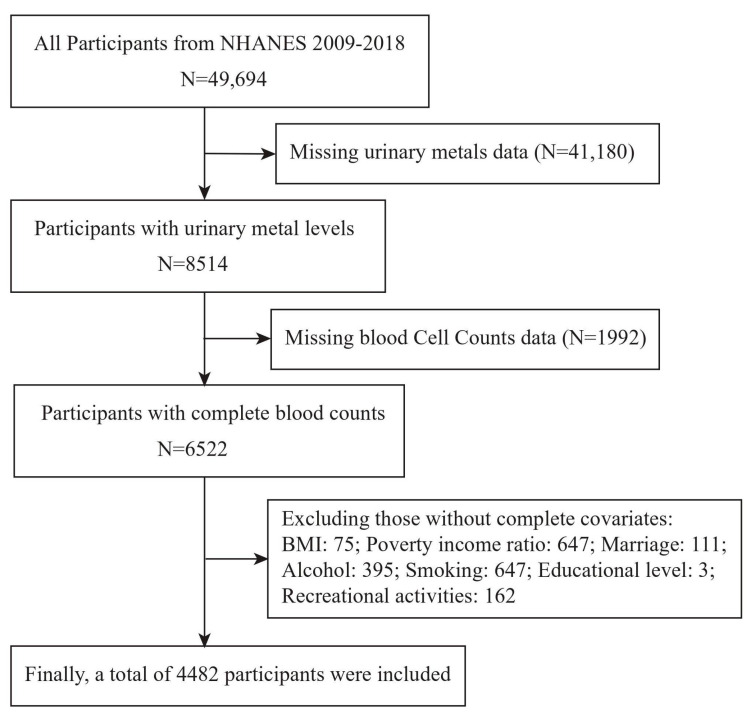
**Flowchart of the participants included in our final analysis (N = 4482), NHANES, USA, 2009–2018.**

The National Health and Nutrition Examination Survey (NHANES) is a cross-sectional population health survey conducted by the Centers for Disease Control and Prevention (CDC) and the National Center for Health Statistics (NCHS) in the United States [23]. A representative sample was selected through multi-stage stratified sampling, with all participants providing informed consent [24]. In this study, data spanning from 2009 to 2018 (five cycles) were obtained from the NHANES official website, comprising a total of 49,694 respondents. Participants lacking laboratory testing information, including those with no metal-related indicators (N = 41,180) and no relevant blood cell measurements (N = 1992), were excluded. To ensure the accuracy of the results, we excluded 2040 participants lacking basic covariate data, such as household income and poverty, race, education, marital status, body mass index (BMI), physical activities, cigarette consumption, and alcohol consumption. Finally, this study included 4482 participants. The participant selection process is depicted in Figure 1.

### 2.2. Measurement of Indicators

The collected urine and blood samples were processed, stored, and transported to appropriate laboratories for analysis. Metal indices were measured using inductively coupled plasma mass spectrometry, including arsenic, dimethylarsinic acid, barium, cadmium, cobalt, cesium, molybdenum, lead, antimony, thallium, tungsten, and mercury. These metals are more realistic, offering complete data, and there were fewer individuals with values below the lower limit of detection or missing. In particular, for metallic arsenic, three specific forms of arsenic (arsenite, arsenic acid, and monomethylarsenic acid) had missing values of more than 80%; so, DMA was used to represent the specific type of arsenic. For values below the lowest detection level (LOD), the heavy metal variables were accounted for as LOD divided by the square root of two. Detailed laboratory methods and experimental procedures can be found on the NHANES website [25]. Hemoglobin, lymphocyte, and platelet values were measured using a hematology-analyzing device (American Beckman Coulter UniCel DxH 800 Analyzer, CA, USA), while serum albumin levels were assessed using Germany Roche modular P and Roche Cobas 6000 chemistry analyzers (Penzberg, Germany) [26]. The immunoinflammatory score indices were calculated with the following formulas: HALP = hemoglobin × albumin × lymphocyte/platelet; SIRI: Neutrophils × monocytes/lymphocytes; AISI: Neutrophils × platelets × monocytes/lymphocytes [15,17,27].

### 2.3. Covariates

Covariate classifications were based on previous studies [28,29,30], including age (20–39 years, 40–59 years, ≥60 years), gender (male and female), race (Mexican American, Other Hispanic, Non-Hispanic White, Non-Hispanic Black, and Other Race-Including Multi-Racial), educational level (Less Than 9th Grade, 9–11th Grade (Includes 12th grade with no diploma), High School Grad/GED or Equivalent, Some College or AA degree, College Graduate or above), marital status (married, widowed, divorced, separated, Never married, Living with a partner), family income to poverty ratio (PIR) categorized as low-income level (<1.3), middle-income level (1.3–3.5), and high-income level (>3.5) [31]. Other covariates included alcohol consumption (No, Yes), smoking (No, Yes), and physical activity (No, Yes). Adult BMI was classified as low (<25 kg/m^2^), medium (25–30 kg/m^2^), and obese (≥30 kg/m^2^) [32].

### 2.4. Statistical Analyses

The demographic characteristics of the participants were described using weighted descriptive statistics, with numbers (n) and percentages (%) for categorical variables and weighted mean and standard deviation (SD) for continuous variables. The calculated blood index and all relevant data of the metal were transformed using natural logarithmic transformation (ln). Subsequently, subgroup analyses were conducted by sex, and all statistical analyses were performed using R4.3.1. Previous studies have demonstrated that in actual living environments, people are often exposed to a variety of mixed pollutants simultaneously, and there exist different association patterns between these environmental pollutants and health outcomes, such as linear growth, plateau type, and inverted U type [33,34]. Therefore, traditional linear models may inadequately capture these relationships or reflect true population exposure. Additionally, pollutants have different chemical structures and biotransformation pathways, and interactions between pollutants and certain covariates (such as sex) may lead to spurious associations. However, if a single environmental pollutant analysis is repeated, that is, using multiple comparisons, it is highly likely to cause false positive errors [35]. Therefore, to assess the combined effect of mixed metal exposure on three inflammatory indices, three statistical models were employed: WQS regression, Q-gcomp, and BKMR models. These models were implemented using the R packages “gWQS”, “Q-gcomp”, and “BKMR”, respectively.

#### 2.4.1. Multiple Linear Regression and Elastic Net Model

Multivariate linear regression models were utilized to examine the correlation between various metal metabolites and human immune inflammation indices. Both metal and immune inflammation indices were treated as continuous variables transformed by ln, with β values and 95% confidence intervals calculated. The multiple linear regression comprised two models: Model 1 was unadjusted for any covariates, whereas Model 2 was adjusted for gender, age, race, educational level, marital status, annual family income, alcohol consumption, smoking status, physical activities, and BMI [30]. Additionally, the elastic net model (ENET), a regularization method combining Ridge regression and Least absolute shrinkage and selection operator (LASSO) regression [31], was employed to identify metal metabolites associated with immune inflammation indices. Furthermore, the corresponding β coefficient was also calculated to quantify the relationship between metal metabolites and immune inflammation indices.

#### 2.4.2. Weighted Quantile Sum Regression Quantile G-Computation Model

WQS regression was used to estimate the combined effects of mixed metal exposures on immune inflammation indices. This approach determines the weight of individual substances in the mixture [36], reducing collinearity and extreme effects of highly correlated pollutants [37]. Furthermore, WQS regression was constructed using two models: one that assumes that the components of the WQS index are all positively correlated with cognitive performance (the positive model), and another that assumes that the components of the WQS index are all negatively correlated with cognitive performance (the negative model) [38]. Additionally, Q-gcomp was also employed to estimate the overall association between metal metabolites and immune inflammation indices. This method combines the simplicity of WQS with the flexibility of G-calculation for analyzing the effects of exposure mixtures [39]. However, unlike the WQS model, Q-gcomp allows each substance to have a positive and negative direction with the immune inflammation indices, and the sum of the weights in the positive and negative directions was 2.

#### 2.4.3. Bayesian Kernel Machine Regression

BKMR was employed to examine the combined effect of mixture heavy metal exposures on three inflammatory indices. This model fixes a mixture of heavy metals at different percentile levels relative to the median and assesses the relative importance of each heavy metal variable on each inflammatory index. BKMR is a non-parametric Bayesian variable selection framework that combines Bayesian and statistical learning methods, including the ability to visualize exposure—responding to different cross sections of the surface—to observe nonlinear associations between monometallic variables and inflammatory indices [40,41]. In this study, the model ran for 10,000 iterations using Markov chain Monte Carlo methods. It was used to estimate the overall health effects of mixtures and assess potential interactions and nonlinear associations between mixed exposures.

## 3. Results

### 3.1. Descriptive Study

Table 1 presents the demographic characteristics of the 4482 surveyed population including household income and poverty, race, education, marital status, and BMI. Among the 4482 participants, 2289 (51.1%) were male, and 1989 (44.4%) were non-Hispanic white, with a weighted age of 48.95 ± 17.90. The mean values of HALP, SIRI, and AISI were 57.19 ± 0.54, 1.24 ± 0.01, and 298.62 ± 3.69, respectively, with inflammatory indices generally higher in males than females. Table S1 contains NHANES codes that help in measuring metals. This is carried out to ensure transparency and reproducibility in the process. Table 2 displays the detection rates and average concentrations of urinary metals in the body. Notably, the detection rate of Sb metal was 66.35%, and the detection rate of other metals exceeded 90%, with metals like As, DMA, Cs, Mo, and Hg reaching 100% detection rates.

### 3.2. Correlations between Metal Alone Exposure and Immunoinflammatory Indexes

**Figure 2:**
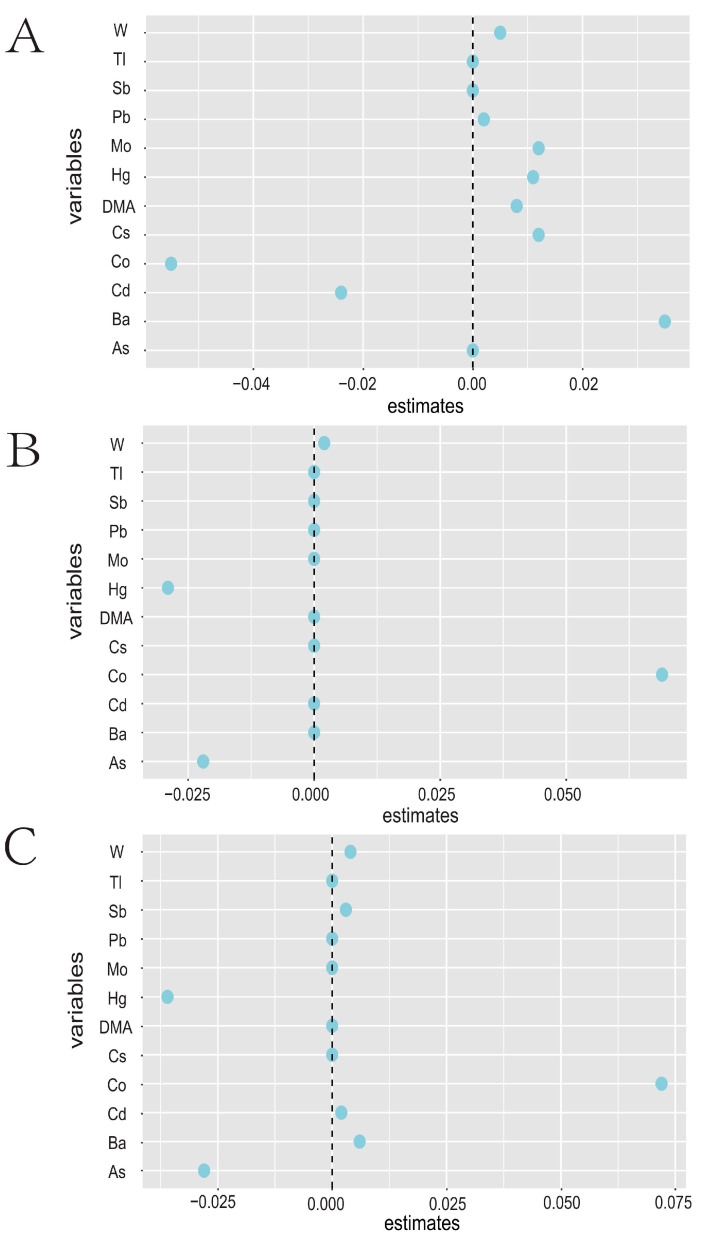
**The correlation coefficients between heavy metal exposure and inflammatory factors were estimated by the elastic net regression model.** The model was adjusted for gender, age, race, educational level, marital status, annual family income, alcohol status, smoking status, physical activity, and BMI. The greater the point deviation from 0, the stronger the correlation between metals exposure and inflammation. (**A**) HALP Index, (**B**) SIRI Index, (**C**) AISI Index.

The correlations between all metals are presented in Figure S1, with R-values ranging between 0.82 and 0.96, indicating strong correlations between metals. Utilizing ENET, we predicted the associations between metal metabolites and immunoinflammatory indicators (Figure 2). Among the 12 metal metabolites, As showed a negative correlation with AISI and SIRI, while Ba exhibited positive correlations with AISI and HALP. Co demonstrated positive correlations with AISI and SIRI, while Co and Cd were negatively correlated with HALP. W showed positive correlations with AISI, SIRI, and HALP.

Table 3 presents the results of linear regression assessing the association between metal metabolites and immunoinflammatory indices. After adjusting for covariates, Ba levels were positively correlated with HALP levels (β: 0.039, 95%CI: 0.025, 0.053), while Cd levels were negatively correlated with HALP levels (β: −0.029, 95%CI: −0.047, −0.012). Co levels were positively correlated with AISI (β: 0.084, 95%CI: 0.050,0.119) and SIRI (β: 0.086, 95%CI: 0.055, 0.116) but negatively correlated with HALP (β: −0.069, 95%CI: −0.089, −0.048). Additionally, Hg levels showed negative correlations with AISI (β: −0.049, 95%CI: −0.077, −0.021) and SIRI (β: −0.039, 95%CI: −0.063, −0.014).

### 3.3. Correlations between Mixture of Metal Exposure and Immunoinflammatory Index

**Figure 3:**
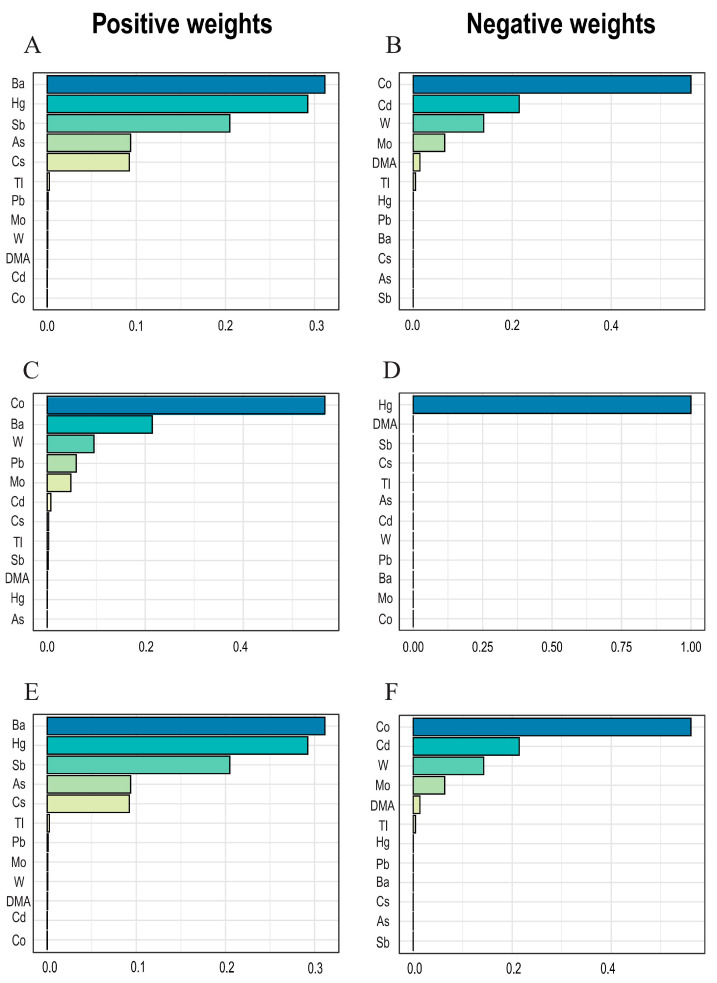
**The WQS model was used to analyze the weights assigned to the effects of metals on inflammatory factors.** The model was adjusted for gender, age, race, educational level, marital status, annual family income, alcohol status, smoking status, physical activity, and BMI. (**A**,**C**,**E**) are the weights of each metal in the positive WQS model, respectively, (**A**) HALP Index, (**C**) SIRI Index, (**E**) AISI Index. (**B**,**D**,**F**) are the weights of each metal in the negative WQS model, respectively, (**B**) HALP Index, (**D**) SIRI Index, (**F**) AISI Index.

The WQS model was employed to estimate the combined effect of a mixture of metal exposure on the immunoinflammatory indices. Figure 3 illustrates that after adjusting for covariates, the Ba levels were positively correlated with HALP and AISI, while the Co levels were positively correlated with SIRI and negatively correlated with HALP and AISI. Moreover, the Hg levels were negatively correlated with SIRI, diverging from lthe inear model and Q-gcomp model results.

**Figure 4:**
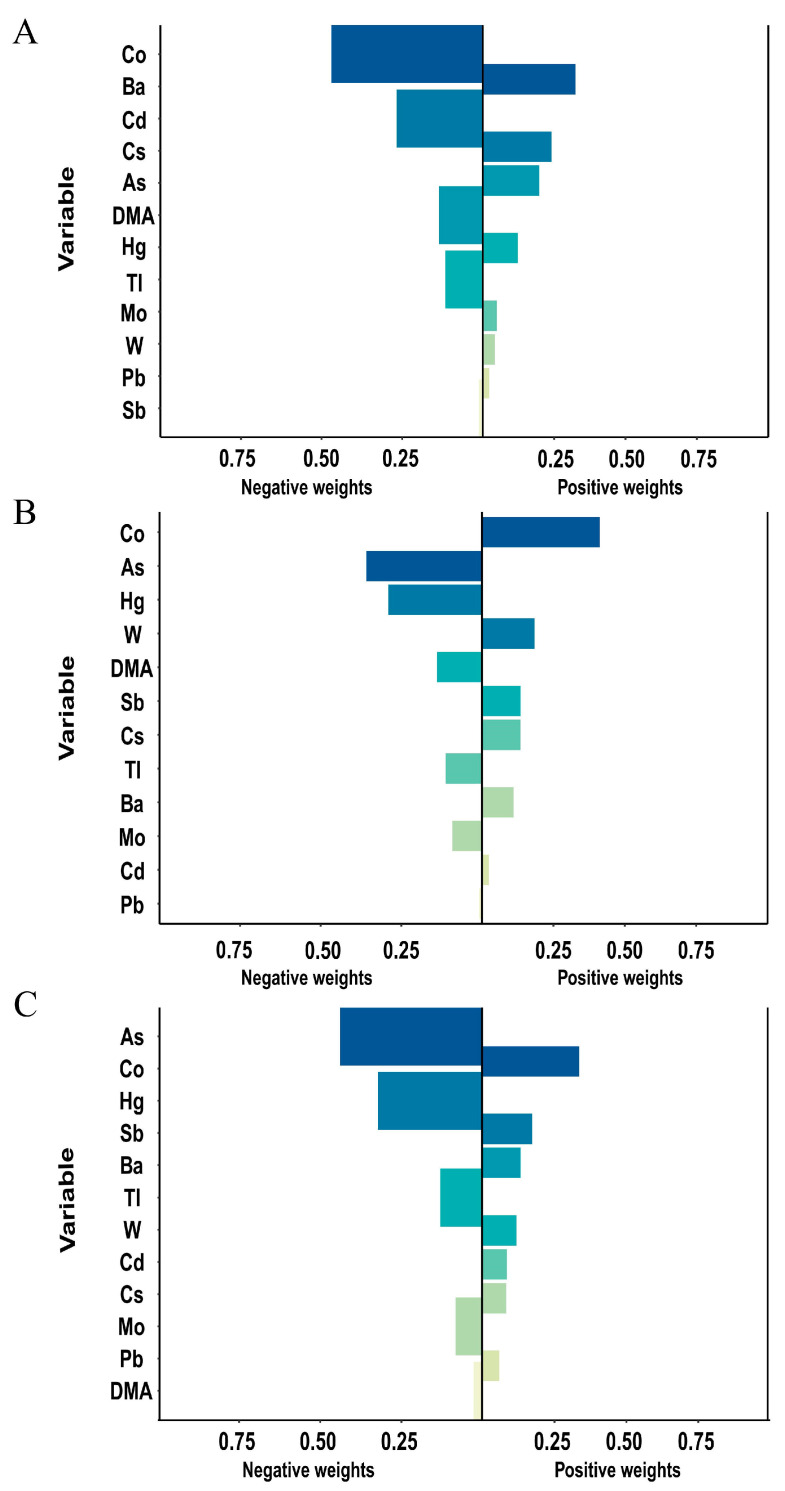
**The directions and magnitude of the assigned weights for each log-transformed metal in relation to inflammation in Q-gcomp.** The model was adjusted for gender, age, race, educational level, marital status, annual family income, alcohol status, smoking status, physical activity, and BMI. (**A**) HALP Index, (**B**) SIRI Index, (**C**) AISI Index.

The Q-gcomp model (Figure 4) demonstrates statistically significant associations between urinary mixture of metal exposure and HALP, AISI, and SIRI levels after adjusting for confounders, aligning with linear model findings. As exhibited negative correlations with AISI and HALP, Ba remained positively correlated with HALP, and Co was positively correlated with AISI and SIRI, while it was negatively correlated with HALP. Co contributed the most to a positive fitting in the Q-gcomp model.

**Figure 5:**
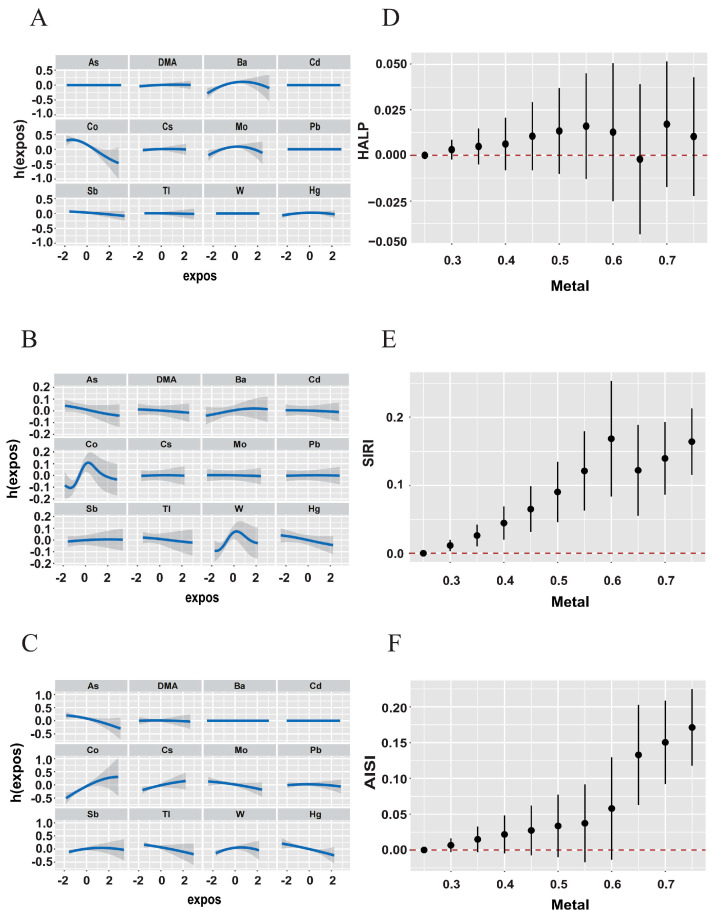
**In the BKMR model, when the concentrations of all other metals were fixed at the median level, the exposure–response relationship function between a single metal and each inflammation index.** (**A**) HALP; (**B**) SIRI; (**C**) AISI. The joint association of mixture exposure of metals in the BKMR model. (**D**) HALP; (**E**) SIRI; (**F**) AISI. The model was adjusted for gender, age, race, educational level, marital status, annual family income, alcohol status, smoking status, physical activity, and BMI.

**Figure 6:**
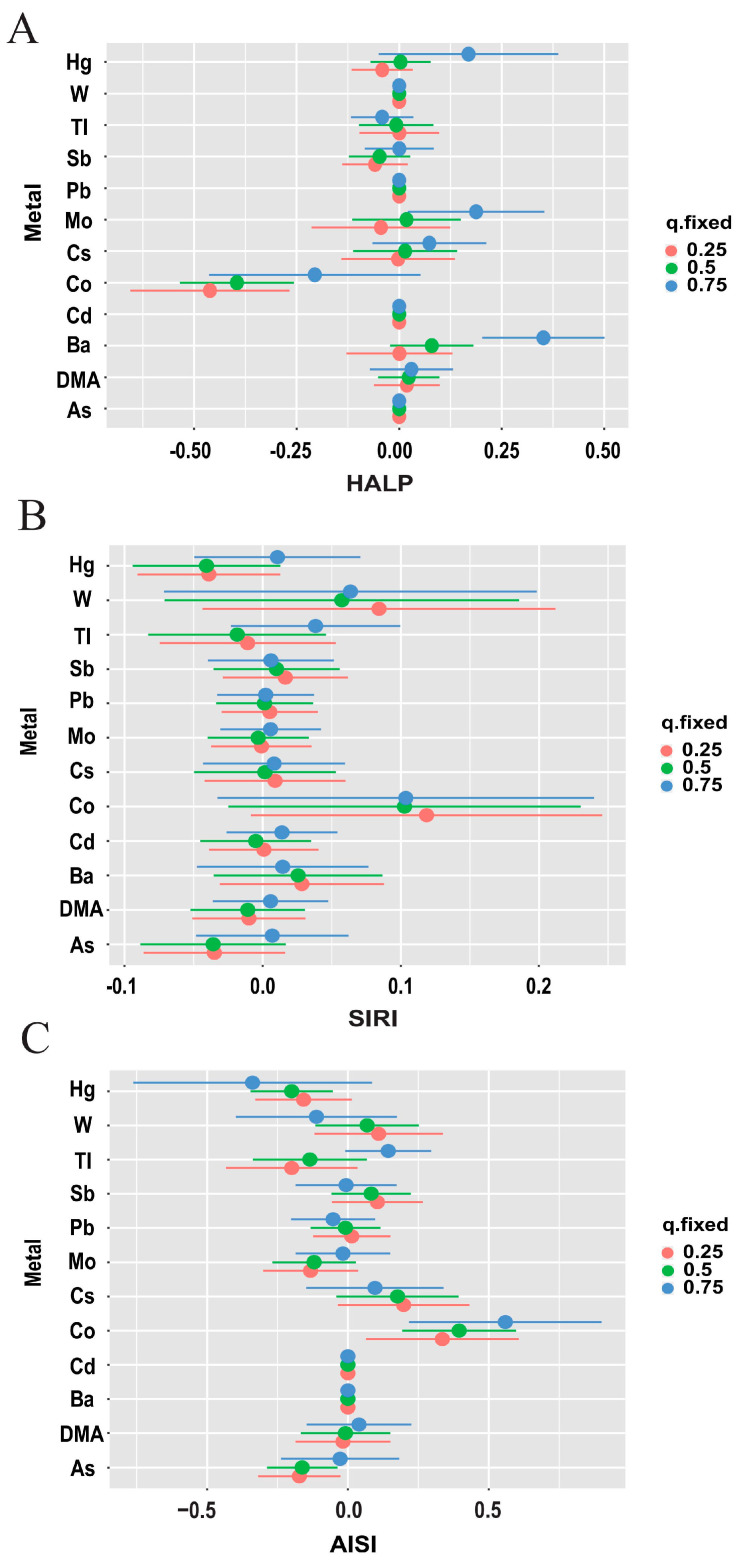
**Estimated differences in inflammation index from 25th to 75th percentiles for each metal when all other metals were fixed at 25th (red line), 50th (green line), or 75th percentile (blue line).** The point represents the estimated value, and the horizontal line represents the 95% confidence interval (CI). (**A**) HALP Index, (**B**) SIRI Index, (**C**) AISI Index. The model was adjusted for age, race, educational level, marital status, annual family income, alcohol status, smoking status, physical activity, and BMI.

The BKMR model reveals significant positive correlations between overall urinary metals and HALP, AISI, and SIRI levels, when all urine metals were above the 30th percentile (Figure 5, Figure 6 and Figure S2). We further analyzed the effect of a single metal variable on the three scores. The correlation trend between individual metal variables and inflammatory indices was observed using a univariate expose–response function curve. In the urine metal model, Ba, Co, As, and Hg were identified as the most influential, with Ba significantly increasing HALP levels, Co significantly increasing AISI and SIRI levels while significantly decreasing HALP levels, and As and Hg significantly reducing AISI and SIRI levels. Additionally, Co exhibited associations with immunoinflammatory indices when other metals were fixed at the median, indicating that As and Co exposure increased SIRI and AISI.

### 3.4. Subgroup Analysis of Urinary Metal and Inflammatory Index

**Figure 7:**
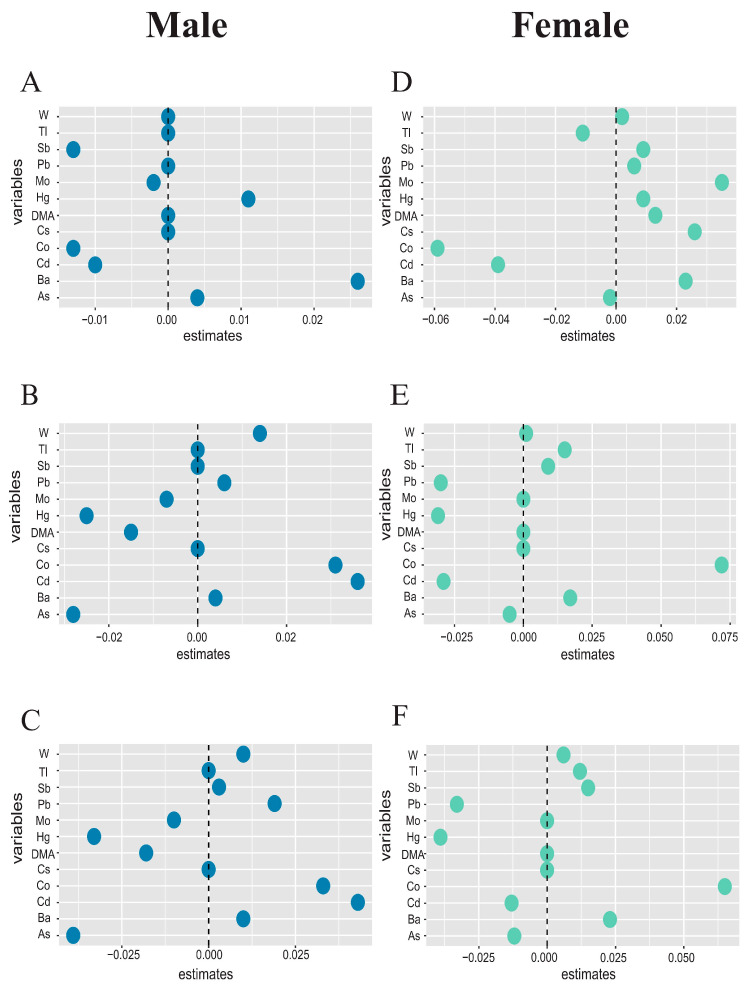
**The correlation coefficients between heavy metal exposure and inflammatory factors were estimated by the elastic net regression model in different genders.** The model was adjusted for age, race, educational level, marital status, annual family income, alcohol status, smoking status, physical activity, and BMI. The greater the point deviation from 0, the stronger the correlation between metals exposure and inflammation. (**A**–**C**) are the estimated coefficients of the association between various metals and inflammation by elastic net regression models in the male population, respectively, (**A**) HALP Index, (**B**) SIRI Index, (**C**) AISI Index. (**D**–**F**) are the estimated coefficients of the association between various metals and inflammation by elastic net regression models in the female population, (**D**) HALP Index, (**E**) SIRI Index, (**F**) AISI Index.

To validate the stability of the results, subgroup analyses explored the effect of metal content in the body on immunoinflammatory indices in different genders. Tables S2 and S3 show the detection rates and average metal concentrations in urine for different genders, respectively, and Figure S3 shows the correlation between all metals for different genders, all in general agreement with the overall results. After stratification by gender, the male multiple regression model shows that As was negatively correlated with AISI, Ba was positively correlated with HALP, and Cd was positively correlated with SIRI (Table S4). Conversely, in the female multiple regression model, Cd was negatively correlated with SIRI, Co was positively correlated with SIRI and AISI while negatively correlated with HALP, and Hg was negatively correlated with AISI (Table S5). Overall, Ba was significantly associated with HALP only in men, while Cd exhibited differing behaviors in SIRI by gender, with men positively correlated and women negatively correlated (i.e., Cd was not associated with SIRI in the general population). Cd was only significantly associated with HALP in women, and Pb was only significantly associated with SIRI in women. Additionally, the elastic network model results grouped by sex generally aligned with the overall grouping results (Figure 7).

Subgroup analyses of WQS and Q-gcomp models for a mixture of metals yielded results consistent with overall findings (Figures S4–S10). Notably, Cd showed a greater association with SIRI and AISI in men, with AISI exhibiting a more significant positive association in men. Among women, Sb emerged as the more influential metal, positively correlated with AISI. Additionally, associations between Pb, Sb, and inflammation markers were more pronounced in women than in men, with women’s HALP scores proving more sensitive to metal exposure. When other metals were fixed at the median, we found that the association between Co and HALP, AISI, and SIRI in the female population remained consistent in the general population. Interestingly, Hg, Pb, Mo, and Ba exposure in women demonstrated heightened sensitivity to immunoinflammatory indicators compared to men.

## 4. Discussion

This study employed various statistical methods to investigate the relationship between metal exposure and immunoinflammatory indices in the adult population of the United States. Recognizing that real-life exposure often involves a simultaneous mixture of multiple pollutants, and traditional analytical strategies may not accurately reflect population exposure or overall health effects, a diverse array of statistical approaches was utilized [42,43]. The findings indicate an association between metal exposure and the immunoinflammatory indices, with potential variations across gender populations, notably with women exhibiting greater sensitivity to the effects of metal exposure on inflammatory indicators than men.

Heavy metals rank among the top 10 priority pollutants contributing to global disease burden and mortality [44]. Prior studies report that exposure to heavy metals, especially Pb and As, is very common in the general population of the United States, aligning with the current study’s results [45,46]. In 2017, the Global Burden of Disease report highlighted that heavy metal exposure contributed to 1.06 million deaths and 24.4 million years of healthy life loss [47]. Heavy metal exposure is particularly associated with cardiovascular disease chronic kidney disease, and other diseases [30,31]. Furthermore, immunoinflammatory response, a post-disease injury response, is of great significance for the identification of early diseases. Accumulating evidence suggests that heavy metal exposure disrupts immune homeostasis and exacerbates inflammation [48]. Inflammation is the body’s protective response to adverse environmental factors, wherein the normal physiological activities of tissues and organs are maintained by eliminating damage-inducing factors, clearing denaturetic and necrotic cells, and initiating repair functions. However, long-term immunoinflammatory activation of the body also contributes to the development of various diseases, including coronary heart disease, pelvic inflammatory disease, cardiovascular disease, and diabetes [13,49].

In this study, both individual and combined metal exposures were correlated with immunoinflammatory markers, with As, Hg, Ba, Cd, and Co playing a major role. Interestingly, As and Hg exhibited significant negative correlations with immune-inflammatory markers. Similarly, Xiaoya et al. [48] reported that Hg was negatively correlated with SII, and mixture analysis revealed Hg as the main substance in the mixture, with negative correlations with SII. Additionally, Tuntinarawat et al. [50] observed that As was found to have a high negative weight on the immune-inflammatory profile, consistent with the results of the present study. Ba has been demonstrated to be associated with oxidative stress and inflammation [51], and a study examining the effect of exposure of mothers to an individual metal and a mixture of metals via the level of inflammation in umbilical cord blood serum reported a positive correlation with interferon-gamma (IFN-γ) and IL-6 [52], along with a similar increase in immune-inflammatory markers in the zygotes. Collectively, the present and prior results demonstrate that Ba induces the dysregulation of the organism through an immune inflammatory response. Moreover, Ba is strongly associated with HALP scores, with Ba accounting for a higher positive weight [53]. Furthermore, the relationship between Ba and HALP was found to be more pronounced in males in the present study, after stratification by gender.

A study found a strong correlation between Cd and Co exposure and HALP scores, and the immunoinflammatory spectrum showed that Cd and Co had a higher negative weight, consistent with this study’s results [53]. One study showed that after adjusting for confounding factors, men had lower levels of metal in their urine than women, highlighting the significant disparity based on gender [54,55]. Moreover, the association between metal exposure and immunoinflammatory markers varied among the gender groups. Additionally, there was a significant negative correlation between Cd and Co exposure and HALP inflammation indicators in women. The mechanism pathway of the metal’s influence on immune inflammation is complex. For instance, Cd, as an industrial and growth toxin, induces oxidative stress by generating free radicals and weakening antioxidant capacity, thus leading to cell necrosis and intracellular inflammatory chemical release along with activating the inflammatory response [7,56]. Co has been reported to induce the inflammatory activation of HaCaT cells, involving the activation of inflammatory bodies and the production of pro-inflammatory cytokines in a dose-dependent manner [57]. Moreover, exposure to cobalt-containing pigments can lead to long-term dysfunction of macrophage function, and in severe cases, long-term inflammation [58]. However, in the present study, Cd was significantly negatively associated with Co exposure. Therefore, we speculate that the changes in Cd and Co concentrations may exert varying effects on the immune inflammatory system, warranting further exploration.

Consistent with previous results, our study did not find an association between Pb exposure and immunoinflammatory indicators, whether it was blood lead or mixture exposure [59]. We speculate that gender differences may play a role in mediating the immunoinflammatory response to metal exposure, possibly due to women’s heightened sensitivity to metal exposure [60]. Several mechanisms can explain this phenomenon. Firstly, exposure to various metals can cause an imbalance in sex hormones in children [61], which, in turn, affects the balance of immune inflammatory response processes [62]. Accordingly, differences in sex hormone levels between genders affect the association between metals and inflammation. Secondly, differences in gene expression between men and women, determined by genetic polymorphism, lead to differences in body sensitivity to metals [63]. Finally, differences in diet and behavior between men and women also affect their exposure to external risk factors [64].

This study has several advantages. Firstly, metals have been demonstrated to induce immunoinflammatory responses. However, there exist only a few studies on the relationship between exposure to heavy metals and immunoinflammatory indicators. To bridge this knowledge gap, this study explored the relationship between multiple metal exposures (both individually and in combination) and immunoinflammatory indicators. Secondly, novel indicators, such as HALP, SIRI, and AISI, were adopted in the selection of immunoinflammatory indicators, which are considered to be more reliable than traditional indicators and can better reflect the immune inflammatory processes [65]. Thirdly, a multi-model analysis of the correlation between mixture heavy metal exposure and immunoinflammatory indices improved the credibility of the results. However, limitations also need to be acknowledged. First, as NHANES survey data were exclusively used, the inferred causal relationship is weak, necessitating further exploration using prospective studies. Second, this study only analyzed the urinary metal content in the body and did not analyze the relationship between blood metal and immune-inflammatory factors. Third, despite efforts to account for many confounding risk factors, residual confounders may exist, highlighting the need for large-scale randomized controlled trials to validate our findings.

## 5. Conclusions

Our study highlights the association between metals and immunoinflammatory markers, underscoring the potential exacerbation of inflammatory responses with a mixture of metal exposure, with gender emerging as a potential influencing factor. Importantly, the adoption of novel immunoinflammatory indicators proved to be more reliable and sensitive in predicting various diseases compared to traditional markers. By elucidating the relationship between metals and immunoinflammatory indicators, our findings offer insights into early detection strategies for metal-induced immune inflammatory responses or diseases, thus providing novel avenues for inflammation prevention efforts.

## Figures and Tables

**Table 1:** **Characteristics of the study population.**

Characteristics N (%)	Total	Male	Female
**Age**	48.95 ± 17.90	48.93 ± 17.98	48.97 ± 17.80
20–39 years	1596 (35.6%)	826 (36.1%)	770 (35.1%)
40–59 years	1391 (31.0%)	692 (30.2%)	699 (31.9%)
≥60 years	1495 (33.4%)	771 (33.7%)	724 (33.0%)
**Race**			
Mexican American	673 (15.0%)	330 (14.4%)	343 (15.6%)
Other Hispanic	439 (9.8%)	223 (9,7%)	216 (9.9%)
Non-Hispanic White	1989 (44.4%)	1010 (44.1%)	979 (44.6%)
Non-Hispanic Black	865 (19.3%)	463 (20.2%)	402 (18.3%)
Other Race—Including Multi-Racial	516 (11.5%)	263 (11.5%)	253 (11.5%)
**Educational level**			
Less Than 9th Grade	440 (9.8%)	234 (10.2%)	206 (9.4%)
9–11th Grade (Includes 12th grade with no diploma)	616 (13.7%)	339 (14.8%)	277 (12.6%)
High School Grad/GED or Equivalent	1053 (23.5%)	580 (25.3%)	473 (21.6%)
Some College or AA degree	1359 (30.3%)	632 (27.6%)	727 (33.2%)
College Graduate or above	1014 (22.6%)	504 (22.0%)	510 (23.3%)
**Marital Status**			
Married	2250 (50.2%)	1242 (52.3%)	1008 (46.0%)
Widowed	350 (7.8%)	92 (4.0%)	258 (11.8%)
Divorced	482 (10.8%)	219 (9.6%)	263 (12.0%)
Separated	142 (3.2%)	69 (3.0%)	73 (3.3%)
Never married	860 (19.2%)	458 (20.0%)	402 (18.3%)
Living with partner	398 (8.9%)	209 (9.1%)	189 (8.6%)
**Ratio of family income to poverty**	2.44 ± 1.60	2.46 ± 1.60	2.41 ± 1.61
**Ratio of family income to poverty**			
<1.3	1486 (33.2%)	735 (32.1%)	751 (34.2%)
1.3–3.5	1718 (38.3%)	897 (39.2%)	821 (37.4%)
>3.5	1278 (28.5%)	657 (28.7%)	621 (28.3%)
**Alcohol drinking**			
No	3381 (75.4%)	1961 (85.7%)	1420 (64.8%)
Yes	1101 (24.6%)	328 (14.3%)	773 (35.2%)
**Smoke**			
No	2046 (45.6%)	1255 (54.8%)	791 (36.1%)
Yes	2436 (54.4%)	1034 (45.2%)	1402 (63.9%)
**Physical activities**			
No	2133 (47.6%)	1134 (49.5%)	999 (45.6%)
Yes	2349 (52.4%)	1155 (50.5%)	1194 (54.4%)
**BMI (kg/m^2^)**			
<25 kg/m^2^	1282 (28.6%)	651 (28.4%)	631 (28.8%)
25 to <30 kg/m^2^	1490 (33.2%)	850 (37.1%)	640 (29.2%)
≥30 kg/m^2^	1710 (38.2%)	788 (34.4%)	922 (42.0%)
**Albumin count (g/dL)**	4.24 ± 0.34	4.33 ± 0.33	4.15 ± 0.33
**Albumin count (g/dL)**	42.43 ± 3.44	43.34 ± 3.31	41.48 ± 3.31
**Lymphocyte count (1000/µL)**	2.15 ± 0.79	2.08 ± 0.83	2.22 ± 0.74
**Monocytes (1000/µL)**	0.55 ± 0.19	0.57 ± 0.19	0.53 ± 0.18
**Lobulated neutral sphere (1000/µL)**	4.31 ± 1.94	4.26 ± 2.10	4.36 ± 1.77
**Hemoglobin (g/dL)**	14.02 ± 1.52	14.84 ± 1.31	13.18 ± 1.24
**Hemoglobin (µmol/L)**	140.23 ± 15.21	148.35 ± 13.10	131.75 ± 12.38
**Platelets (µmol/L)**	237.88 ± 61.57	225.37 ± 55.90	250.94 ± 64.47
**HALP score**	57.19 ± 36.12	62.54 ± 32.22	51.60 ± 39.03
**SIRI index**	1.24 ± 0.89	1.32 ± 0.95	1.15 ± 0.82
**AISI index**	298.62 ± 247.31	302.74 ± 245.83	294.32 ± 248.88

Note: BMI is body mass index.

**Table 2:** **The distribution of the urinary metabolites in the study population.**

Metal (µg/L)	Detection Rate N (%)	Mean	Percentiles				
			P5	P25	P50	P75	P95
As	100.00	4.91	1.55	3.05	5.40	6.42	7.87
DMA	100.00	4.45	1.09	2.53	4.91	5.81	6.72
Ba	99.44	4.16	0.90	3.43	4.41	5.26	6.39
Cd	91.32	3.06	1.31	2.37	3.08	3.77	4.72
Co	99.53	3.66	2.24	3.11	3.70	4.23	5.09
Cs	100.00	4.59	1.15	2.11	5.47	6.14	6.78
Mo	100.00	6.15	3.08	3.90	6.77	8.15	9.19
Pb	97.57	3.43	1.79	2.83	3.47	4.04	5.07
Sb	66.35	1.87	0.47	1.28	1.76	2.37	3.62
Tl	94.91	4.08	1.86	3.18	3.97	4.88	6.42
W	90.12	2.65	1.48	2.20	2.69	3.12	3.71
Hg	100.00	2.12	1.42	2.20	2.20	2.20	2.20

**Table 3:** **The relationship between mixture exposure of metal metabolites and immune inflammation index.**

Metal	HALP Score	SIRI Index	AISI Index
	β (95%CI)	β (95%CI)	β (95%CI)
**Model 1**			
As	−0.015 (−0.021, 0.015)	−0.018 (−0.044, 0.008)	**−0.035 (−0.064, −0.006) ^a^**
DMA	0.009 (−0.017, 0.034)	−0.036 (−0.074, 0.001)	−0.025 (−0.067, 0.017)
Ba	**0.040 (0.026, 0.055) ^a^**	0.001 (−0.020, 0.022)	0.012 (−0.012, 0.035)
Cd	**−0.052 (−0.068, −0.036) ^a^**	**0.033 (0.010, 0.056) ^a^**	**0.033 (0.008, 0.059) ^a^**
Co	**−0.094 (−0.115, 0.073)** **^a^**	**0.054 (0.024, 0.084) ^a^**	**0.073 (0.040, 0.107) ^a^**
Cs	0.028 (−0.001, 0.056)	−0.014 (−0.055, 0.028)	−0.011 (−0.058, 0.035)
Mo	**0.028 (0.009, 0.048) ^a^**	−0.008 (−0.037, 0.021)	−0.019 (−0.051, 0.013)
Pb	**0.026 (0.007, 0.046) ^a^**	**0.028 (0.000, 0.057) ^a^**	0.002 (−0.029, 0.034)
Sb	0.013 (−0.005, 0.032)	0.001 (−0.026, 0.028)	0.014 (−0.016, 0.044)
Tl	−0.005 (−0.022, 0.011)	0.012 (−0.012, 0.036)	0.010 (−0.016, 0.037)
W	**0.019 (0.001, 0.036)** **^a^**	0.011 (−0.014, 0.037)	**0.017 (−0.011, −0.046)** **^a^**
Hg	−0.005 (−0.022, 0.012)	**−0.051 (−0.076, −0.027)** **^a^**	−0.060 (−0.088, 0.033)
**Model 2**			
As	−0.001 (−0.019, 0.017)	−0.021 (−0.046, 0.005)	**−0.032 (−0.062, −0.003) ^a^**
DMA	0.011 (−0.014, 0.037)	−0.012 (−0.049, 0.025)	−0.006 (−0.048, 0.036)
Ba	**0.039 (0.025, 0.053) ^a^**	0.004 (−0.017, 0.025)	0.010 (−0.013, 0.034)
Cd	**−0.029 (−0.047, −0.012) ^a^**	0.002 (−0.024, 0.028)	0.014 (−0.015, 0.043)
Co	**−0.069 (−0.089, −0.048) ^a^**	**0.086 (0.055, 0.116) ^a^**	**0.084 (0.050, 0.119) ^a^**
Cs	0.023 (−0.005, 0.051)	−0.015 (−0.056, 0.026)	−0.013 (−0.059, 0.034)
Mo	0.016 (−0.004, 0.035)	−0.016 (−0.045, 0.012)	−0.021 (−0.053, 0.011)
Pb	0.008 (−0.012, 0.028)	−0.016 (−0.046, 0.013)	−0.010 (−0.043, 0.023)
Sb	−0.005 (−0.023, 0.014)	0.004 (−0.023, 0.031)	0.010 (−0.020, 0.040)
Tl	−0.005 (−0.021, 0.012)	0.012 (−0.012, 0.036)	0.008 (−0.019, 0.034)
W	0.008 (−0.051, 0.025)	0.019 (−0.006, 0.045)	0.018 (−0.010, 0.047)
Hg	0.013 (−0.003, 0.030)	**−0.039 (−0.063, −0.014) ^a^**	**−0.049 (−0.077, −0.021) ^a^**

^a^: Significant results are in bold; Model I: Models without covariate adjustment; Model II: Models adjusted by covariates; the covariates are age, race, educational level, marital status, annual family income, alcohol status, smoking status, physical activity, and BMI.

## Data Availability

The data were retrieved from publicly available resources and can be accessed from National Center for Health Statistics of Center for Disease Control and Prevention through https://www.cdc.gov/nchs/nhanes/index.htm, accessed on 11 March 2024.

## Supplementary Materials

The following supporting information can be downloaded at: https://www.mdpi.com/article/10.3390/toxics12050316/s1, Table S1. NHANES codes for urine metal measurements; Table S2. The distribution of the urinary metabolites in the study population (Male); Table S3. The distribution of the urinary metabolites in the study population (Female); Table S4. The relationship between mixed exposure of metal metabolites and immune inflammation index (Male); Table S5. The relationship between mixed exposure of metal metabolites and immune inflammation index (Female); Figure S1. Pearson correlation between the logarithmic concentrations of 12 metals (N = 4482), NHANES, USA, 2009–2018; Figure S2. In Bayesian kernel regression, the relationship between each metal and inflammation index. This figure is a bivariate exposure response function of metal exposure and inflammation index when a metal is fixed in different (25th, 50th, 75th) percentiles and other metals are fixed at the 50th percentile, the average difference between the other metal and the inflammation index as a bivariate exposure response function. (A) HALP Index, (B) SIRI Index, (C) AISI Index. The model was adjusted for gender, age, race, educational level, marital status, annual family income, alcohol status, smoking status, physical activity, and BMI; Figure S3. Pearson correlation between the logarithmic concentrations of 12 metals for different sexes, (A) Male, (B) Female; Figure S4. The weights of each metal in the WQS model regression index in the male population. The model was adjusted for age, race, educational level, marital status, annual family income, alcohol status, smoking status, physical activity, and BMI. (A–C) are the weights of each metal in the positive WQS model, respectively, (A) HALP Index, (B) SIRI Index, (C) AISI Index. (D–F) are the weights of each metal in the negative WQS model, (D) HALP Index, (E) SIRI Index, (F) AISI Index; Figure S5. The weights of each metal in the WQS model regression index in the female population. The model was adjusted for age, race, educational level, marital status, annual family income, alcohol status, smoking status, physical activity, and BMI. (A,C,D) are the weights of each metal in the positive WQS model, respectively, (A) HALP Index, (C) SIRI Index, (D) AISI Index. (B) is the weights of each metal in the negative WQS model, (B) HALP Index; Figure S6. The weight of each metal in the Q-gcomp model index in the male and female groups. (A–C) are the directions and magnitude of the assigned weights for each log-transformed metal in relation to inflammation in male population in quantile g-computation. (A) HALP Index, (B) SIRI Index, (C) AISI Index. (D–F) are the directions and magnitude of the assigned weights for each log-transformed metal in relation to inflammation in female population in quantile g-computation. (D) HALP Index, (E) SIRI Index, (F) AISI Index. The model was adjusted for age, race, educational level, marital status, annual family income, alcohol status, smoking status, physical activity, and BMI; Figure S7. In the BKMR model, when the concentrations of all other metals were fixed at the median level, the exposure–response relationship function between a single metal and each inflammation index. (A–C) are the relationships in male population, (A) HALP Index, (B) SIRI Index, (C) AISI Index. (D–F) are the relationships in female population, (D) HALP Index, (E) SIRI Index, (F) AISI Index. The model was adjusted for age, race, educational level, marital status, annual family income, alcohol status, smoking status, physical activity, and BMI; Figure S8. The joint association of mixed exposure of metal in the BKMR model. (A–C) are the relationships in male population, (A) HALP Index, (B) SIRI Index, (C) AISI Index. (D–F) are the relationships in female population, (D) HALP Index, € SIRI Index, (F) AISI Index. The model was adjusted for age, race, educational level, marital status, annual family income, alcohol status, smoking status, physical activity, and BMI; Figure S9. This figure describes the estimated difference in inflammation index for each metal from the 25th to the 75th percentile when all other metals are fixed at the 25th (red line), 50th (green line), or 75th percentile (blue line). The point represents the estimated value, and the horizontal line represents the 95% confidence interval (CI). (A–C) are the relationships in male population, (A) HALP Index, (B) SIRI Index, (C) AISI Index. (D–F) are the relationships in female population, (D) HALP Index, (E) SIRI Index, (F) AISI Index. The model was adjusted for age, race, educational level, marital status, annual family income, alcohol status, smoking status, physical activity, and BMI; Figure S10. This figure is a bivariate exposure response function of metal exposure and inflammation index when a metal is fixed in different (25th, 50th, 75th) percentiles and other metals are fixed at the 50th percentile, the average difference between the other metal and the inflammation index as a bivariate exposure response function. (A–C) are the relationships in male population, (A) HALP Index, (B) SIRI Index, (C) AISI Index. (D–F) are the relationships in female population, (D) HALP Index, (E) SIRI Index, (F) AISI Index. The model was adjusted for age, race, educational level, marital status, annual family income, alcohol status, smoking status, physical activity, and BMI.
