# Supplementary material for: Association of Metal Exposure with Novel Immunoinflammatory Indicators
Source: Toxics. 2024 Apr 26;12(5):316. doi: 10.3390/toxics12050316 (PMC11125449; doi:10.3390/toxics12050316)
Supplement: Supplementary file 1 [file toxics-12-00316-s001.zip › toxics-2928943-supplementary.pdf]

Association of metal exposure with novel immunoinflammatory indicators

CATALOG

Table S1....1
Table S2....2
Table S3....3
Table S4....4
Table S5....5
Figure S1....6
Figure S2....7
Figure S3....8
Figure S4....9
Figure S5....10
Figure S6....11
Figure S7....12
Figure S8....13
Figure S9....14
Figure S10....15

Table S1. NHANES codes for urine metal measurements

Urine measuring metal	Name of code (µg/L)
As	URXUAS
DMA	URXUDMA
Ba	URXUBA
Cd	URXUCD
Co	URXUCO
Cs	URXUCS
Mo	URXUMO
Pb	URXUPB
Sb	URXUSB
Tl	URXUTL
W	URXUW
Hg	URXUHG

Table S2. The distribution of the urinary metabolites in the study population. (Male)

Metal	Detection rate N (%)	Mean	Percentiles				
			P5	P25	P50	P75	P95
As	100.00	4.97	1.50	3.00	5.57	6.45	7.82
DMA	100.00	4.52	1.03	2.66	4.91	5.88	6.74
Ba	99.30	4.27	0.96	3.61	4.49	5.31	6.51
Cd	91.57	3.07	1.39	2.39	3.11	3.78	4.73
Co	99.65	3.63	2.29	3.11	3.67	4.13	4.78
Cs	100.00	4.67	1.06	2.15	5.58	6.19	6.77
Mo	100.00	6.20	3.02	3.83	6.98	8.30	9.28
Pb	98.47	3.58	1.91	3.00	3.64	4.19	5.15
Sb	71.52	1.98	0.47	1.41	1.87	2.42	3.66
Tl	95.15	4.11	1.86	3.18	4.01	4.88	6.46
W	91.87	2.68	1.55	2.25	2.74	3.10	3.70
Hg	100.00	2.14	2.20	2.20	2.20	2.20	2.20

Table S3. The distribution of the urinary metabolites in the study population. (Female)

Metal	Detection rate N (%)	Mean	Percentiles				
			P5	P25	P50	P75	P95
As	100.00	4.84	1.60	3.11	5.22	6.32	7.94
DMA	100.00	4.39	1.13	2.44	4.91	5.70	6.66
Ba	99.59	4.05	0.87	3.22	4.28	5.20	6.28
Cd	91.06	3.04	0.92	2.36	3.06	3.76	4.72
Co	99.41	3.69	2.16	3.10	3.71	4.36	5.23
Cs	100.00	4.51	1.25	2.09	5.35	6.06	6.78
Mo	100.00	6.09	3.18	3.97	6.62	8.02	9.02
Pb	96.63	3.25	1.61	2.64	3.30	3.87	4.84
Sb	60.97	1.74	0.47	1.13	1.61	2.22	3.57
Tl	94.66	4.04	1.86	3.14	3.95	4.90	6.22
W	88.28	2.61	1.40	2.08	2.63	3.15	3.74
Hg	100.00	2.11	1.13	2.20	2.20	2.20	2.20

Table S4. The relationship between mixed exposure of metal metabolites and immune inflammation index. (Male)

Metal	HALP score β (95%CI)	SIRI index β (95%CI)	AISI index β (95%CI)
**Model 1**			
As	-0.022 (-0.030, 0.022)	-0.026 (-0.063, 0.012)	**-0.044 (-0.086, -0.002)**[a]
DMA	0.013 (-0.023, 0.050)	-0.053 (-0.106, 0.001)	-0.045 (-0.104, 0.015)
Ba	**0.048 (0.029, 0.068)**[a]	-0.038 (-0.039, 0.019)	0.013 (-0.029, 0.036)
Cd	**-0.043 (-0.065, -0.022)**[a]	**0.102 (0.070, 0.134)**[a]	**0.085 (0.049, 0.121)**[a]
Co	**-0.043 (-0.077, -0.010)**[a]	**0.071 (0.022, 0.120)**[a]	**0.063 (0.008, 0.118)**[a]
Cs	0.027 (-0.035, 0.045)	-0.049 (-0.071, 0.044)	-0.004 (-0.066, 0.064)
Mo	-0.029 (-0.033, 0.022)	-0.034 (-0.074, 0.006)	-0.031 (-0.076, 0.014)
Pb	-0.008 (-0.033, 0.018)	0.035 (-0.003, 0.073)	0.038 (-0.005, 0.081)
Sb	0.011 (-0.023, 0.027)	-0.021 (-0.057, 0.015)	0.000 (-0.041, 0.041)
Tl	0.005 (-0.017, 0.027)	-0.003 (-0.033, 0.032)	-0.040 (-0.046, 0.027)
W	0.021 (-0.003, 0.046)	0.028 (-0.029, 0.042)	0.037 (-0.030, 0.050)
Hg	0.024 (-0.018, 0.030)	**-0.040 (-0.075, -0.004)**[a]	**-0.050 (-0.090, -0.010)**[a]
**Model 2**			
As	0.009 (-0.017, 0.034)	-0.031 (-0.068, 0.006)	**-0.043 (-0.085, 0.000)**[a]
DMA	-0.009 (-0.038, 0.034)	-0.026 (-0.078, 0.027)	-0.028 (-0.088, 0.031)
Ba	**0.039 (0.019, 0.058)**[a]	0.009 (-0.027, 0.089)	0.026 (-0.025, 0.040)
Cd	-0.014 (-0.039, -0.011)	**0.048 (0.012, 0.084)**[a]	**0.055 (0.014, 0.096)**[a]
Co	**-0.037 (-0.070, -0.003)**[a]	**0.054 (0.006, 0.102)**[a]	**0.053 (-0.002, 0.107)**[a]
Cs	0.023 (-0.035, 0.044)	-0.019 (-0.062, 0.052)	0.014 (-0.060, 0.069)
Mo	-0.008 (-0.036, 0.019)	-0.032 (-0.072, 0.007)	-0.031 (-0.075, 0.014)
Pb	0.029 (-0.021, 0.032)	0.028 (-0.031, 0.047)	0.025 (-0.019, 0.070)
Sb	-0.023 (-0.048, 0.002)	-0.004 (-0.038, 0.036)	0.016 (-0.037, 0.046)
Tl	0.005 (-0.016, 0.027)	-0.004 (-0.033, 0.031)	-0.043 (-0.046, 0.026)
W	0.007 (-0.017, 0.032)	0.030 (-0.005, 0.066)	0.021 (-0.019, 0.062)
Hg	0.018 (-0.007, 0.042)	**-0.037 (-0.072, -0.002)**[a]	**-0.044 (-0.084, -0.004)**[a]

[a]:Significant results were in bold; Model I: Models without covariate adjustment; Model II: Models Adjusted by Covariates, The covariates are age, race, educational level, marital status, annual family income, alcohol status, smoking status, physical activities and BMI.

Table S5. The relationship between mixed exposure of metal metabolites and immune inflammation index. (Female)

Metal	HALP score<br>β (95%CI)	SIRI index<br>β (95%CI)	AISI index<br>β (95%CI)
**Model 1**			
As	**-0.013(-0.037, -0.011)** [a]	-0.020 (-0.056, 0.015)	-0.030 (-0.070, 0.010)
DMA	0.012 (-0.023, 0.048)	-0.039 (-0.064, 0.041)	-0.010 (-0.062, 0.056)
Ba	**0.025 (0.004, 0.046)** [a]	0.020 (-0.011, 0.050)	0.028 (-0.006, 0.063)
Cd	**-0.033 (-0.055, -0.011)** [a]	-0.013 (-0.046, 0.019)	-0.031 (-0.047, 0.026)
Co	**-0.076 (-0.102, -0.049)** [a]	**0.081 (0.041, 0.120)** [a]	**0.090 (0.046, 0.134)** [a]
Cs	0.039 (-0.001, 0.079)	-0.060 (-0.076, 0.041)	-0.053 (-0.083, 0.049)
Mo	**0.043 (0.016, 0.071)** [a]	0.019 (-0.035, 0.046)	-0.017 (-0.052, 0.040)
Pb	0.012 (-0.017, 0.041)	-0.041 (-0.084, 0.002)	**-0.067 (-0.116, -0.019)** [a]
Sb	0.018 (-0.009, 0.046)	0.019 (-0.022, 0.059)	0.031 (-0.015, 0.076)
Tl	-0.021 (-0.045, 0.003)	0.026 (-0.009, 0.061)	0.032 (-0.007, 0.072)
W	0.004 (-0.020, 0.028)	0.018 (-0.031, 0.040)	0.018 (-0.022, 0.058)
Hg	0.004 (-0.018, 0.027)	**-0.041 (-0.074, -0.007)** [a]	**-0.059 (-0.097, -0.021)** [a]
**Model 2**			
As	-0.008 (-0.033, 0.016)	-0.037 (-0.047, 0.025)	**-0.022 (-0.062, 0.018)** [a]
DMA	0.021 (-0.015, 0.057)	0.018 (-0.047, 0.058)	0.061 (-0.041, 0.077)
Ba	**0.026 (0.006, 0.047)** [a]	0.021 (-0.010, 0.051)	0.027 (-0.007, 0.062)
Cd	**-0.046 (-0.071, -0.022)** [a]	**-0.041 (-0.077, -0.004)** [a]	-0.024 (-0.064, 0.017)
Co	**-0.076 (-0.103, -0.049)** [a]	**0.088 (0.049, 0.128)** [a]	**0.092 (0.048, 0.137)** [a]
Cs	**0.042 (0.002, 0.083)** [a]	-0.073 (-0.080, 0.038)	-0.025 (-0.092, 0.041)
Mo	**0.044 (0.016, 0.072)** [a]	0.012 (-0.037, 0.045)	-0.008 (-0.054, 0.038)
Pb	0.011 (-0.020, 0.041)	**-0.051 (-0.095, -0.007)** [a]	-0.059 (-0.109, -0.009)
Sb	0.013 (-0.015, 0.040)	0.017 (-0.023, 0.058)	0.022 (-0.023, 0.068)
Tl	-0.020 (-0.044, 0.004)	0.028 (-0.007, 0.064)	0.028 (-0.011, 0.068)
W	0.002 (-0.023, 0.026)	0.028 (-0.029, 0.042)	0.012 (-0.028, 0.052)
Hg	0.010 (-0.013, 0.034)	**-0.038 (-0.072, -0.004)** [a]	**-0.051 (-0.089, -0.013)** [a]

*Significant results were in bold; Model I: Models without covariate adjustment; Model II: Models Adjusted by Covariates, The covariates are age, race, educational level, marital status, annual family income, alcohol status, smoking status, physical activities and BMI.

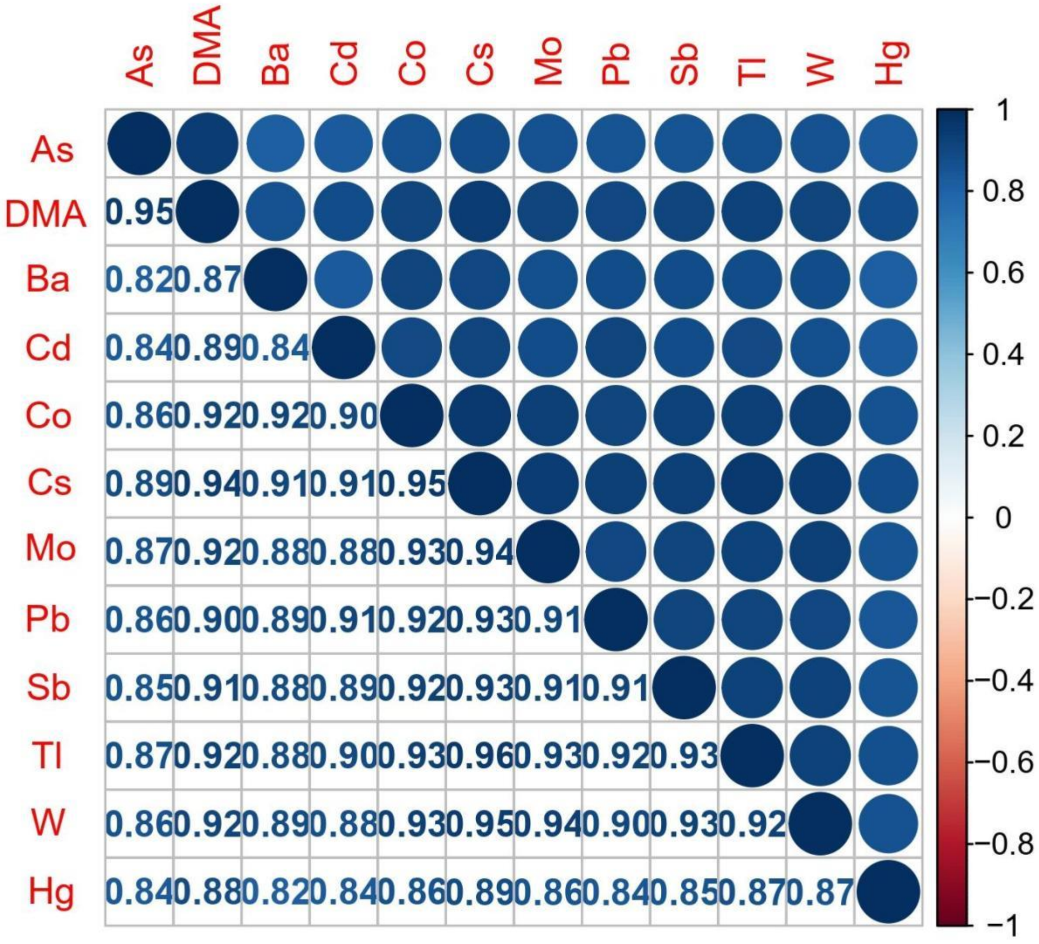

Figure S1. Pearson correlation between the logarithmic concentrations of 12 metals, (N=4482), NHANES, USA, 2009–2018.

A

B

C

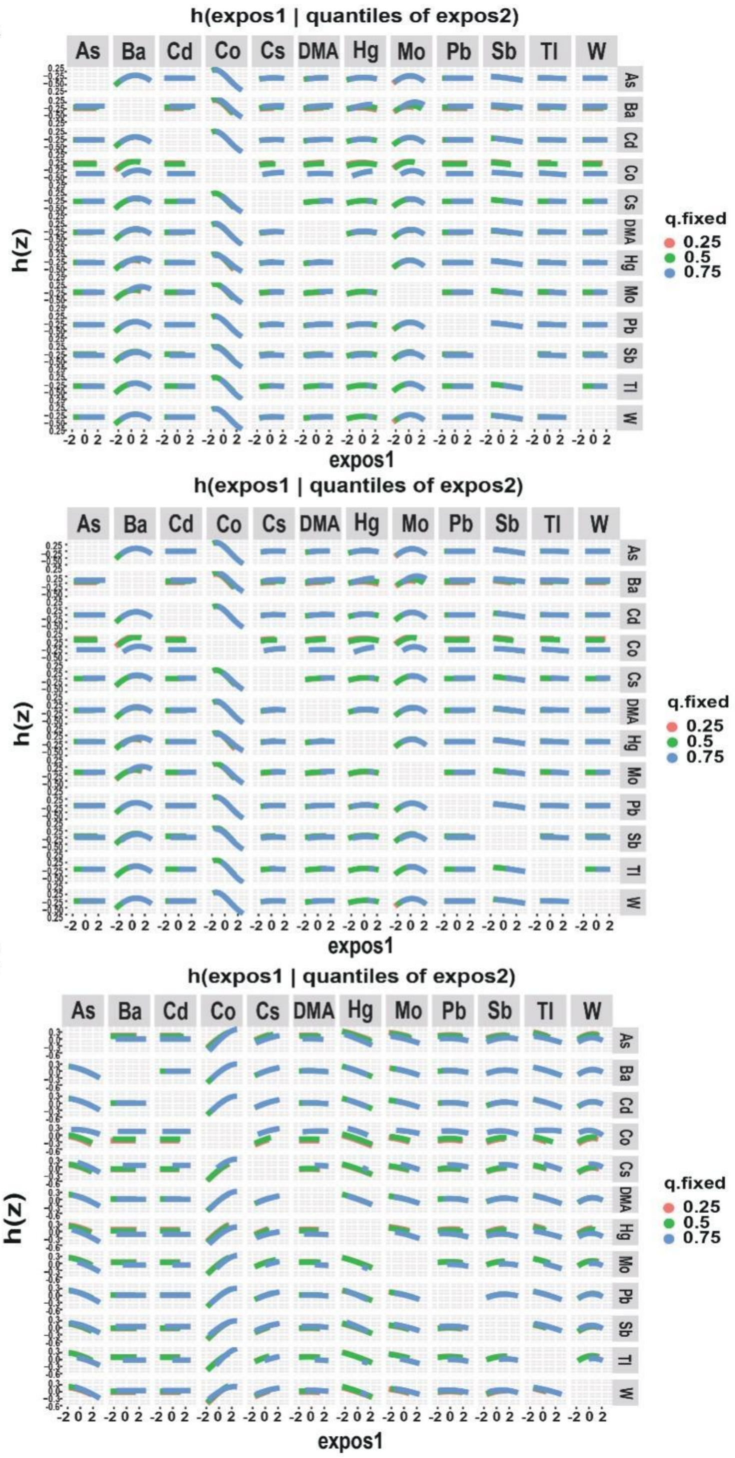

Figure S2. In BKMR regression, the relationship between each metal and inflammation index. This figure is a bivariate exposure response function of metal exposure and inflammation index when a metal is fixed in different (25th , 50th ,75th) percentiles and other metals are fixed at the 50th percentile, the average difference between the other metal and the inflammation index as a bivariate exposure response function. (**A**) HALP Index, (**B**) SIRI Index, (**C**) AISI Index. The model was adjusted for gender, age, race, educational level, marital status, annual family income, alcohol status, smoking status, physical activities and BMI.

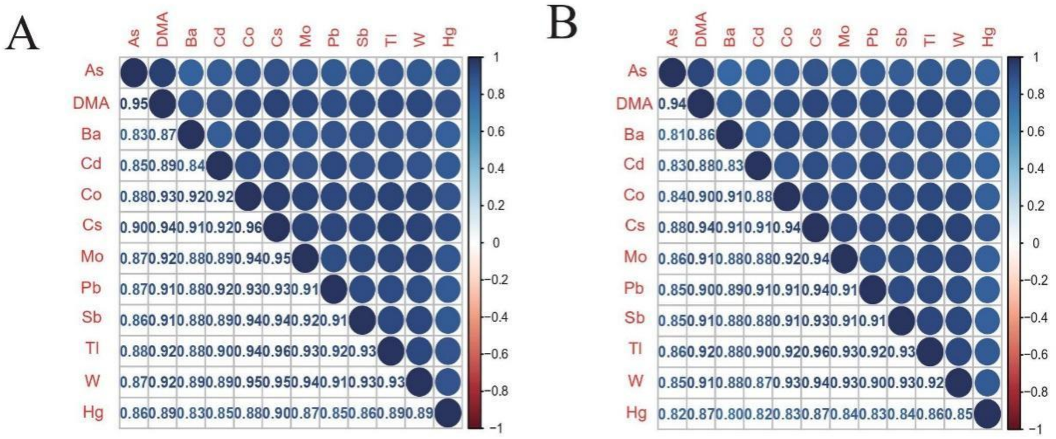

Figure S3. Pearson correlation between the logarithmic concentrations of 12 metals for different sexes. (**A**) Male, (**B**) Female.

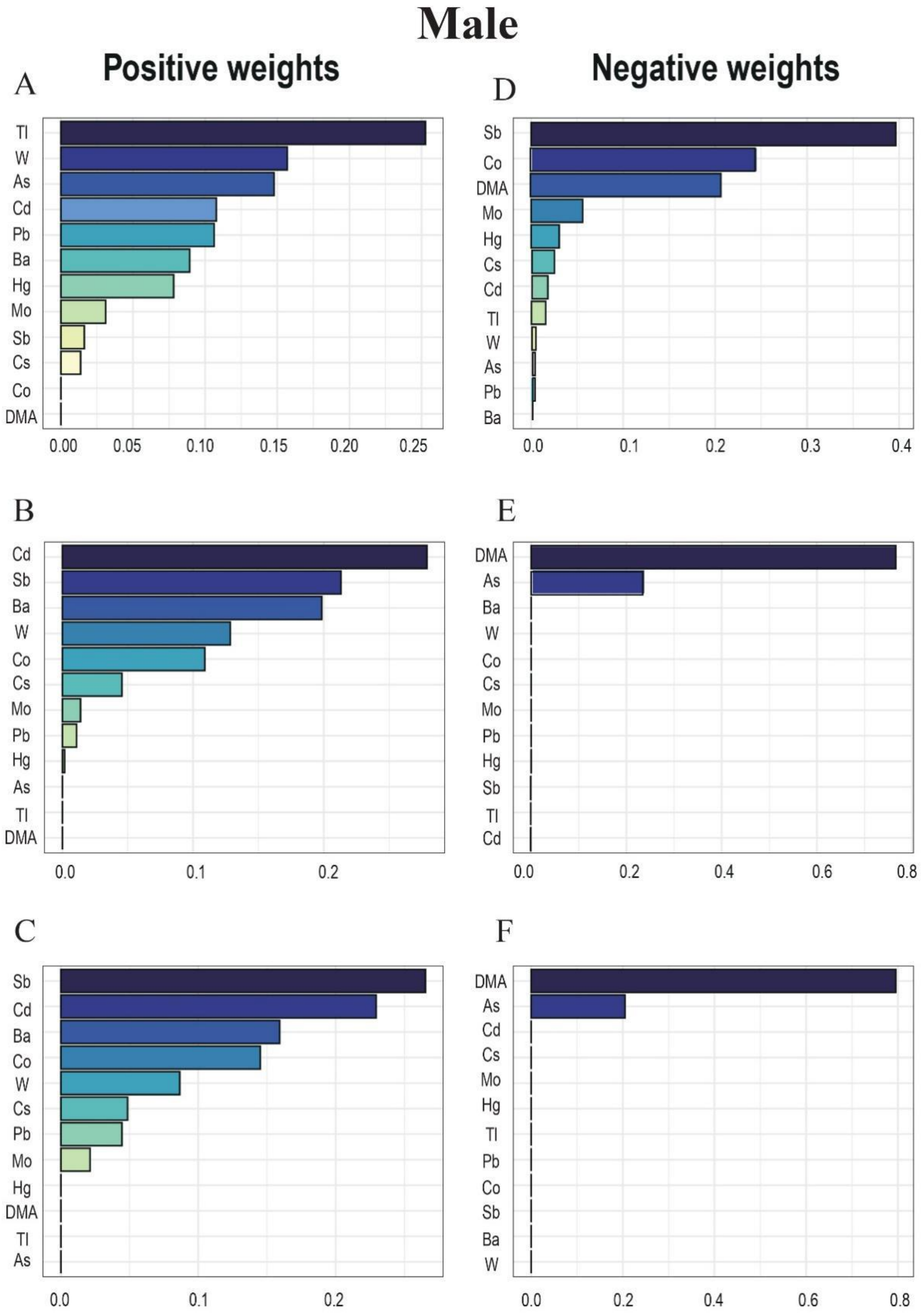

Figure S4. The weights of each metal in the WQS model regression index in the male population. The model was adjusted for age, race, educational level, marital status, annual family income, alcohol status, smoking status, physical activities and BMI. (**A**–**C**) are the weights of each metal in the positive WQS model, respectively, (**A**) HALP Index, (**B**) SIRI Index, (**C**) AISI Index. (**D**–**F**) are the weights of each metal in the negative WQS model, (**D**) HALP Index, (**E**) SIRI Index, (**F**) AISI Index.

Female

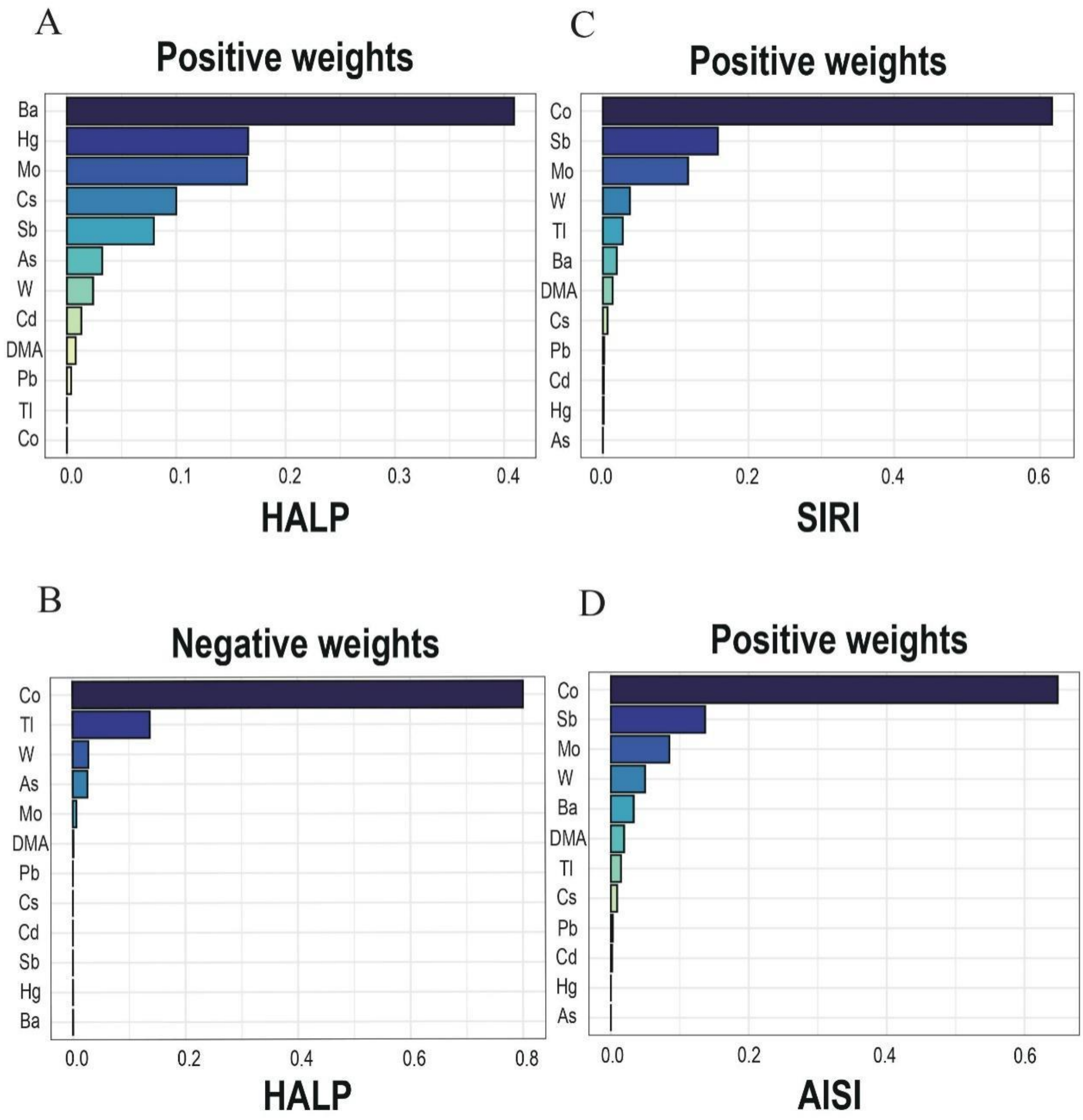

Figure S5. The weights of each metal in the WQS model regression index in the female population. The model was adjusted for age, race, educational level, marital status, annual family income, alcohol status, smoking status, physical activities and BMI. (**A**,**C**,**D**) are the weights of each metal in the positive WQS model, respectively, (**A**) HALP Index, (**C**) SIRI Index, (**D**) AISI Index. (**B**) is the weights of each metal in the negative WQS model, (**B**) HALP Index.

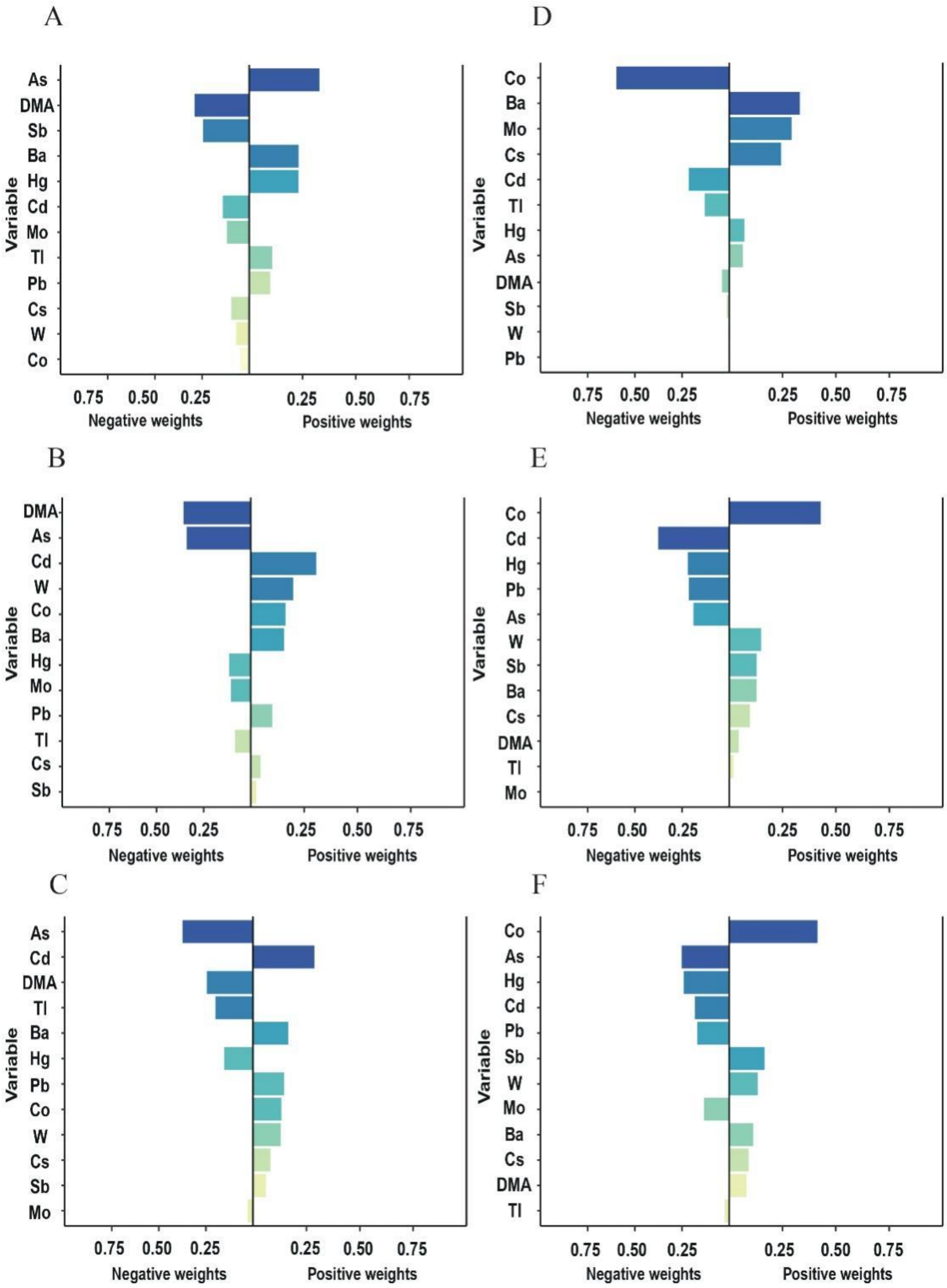

Figure S6. The weight of each metal in the Q-gcomp model index in the male and female groups. (**A**–**C**) are the directions and magnitude of the assigned weights for each log-transformed metal in relation to inflammation in male population in quantile g-computation. (**A**) HALP Index, (**B**) SIRI Index, (**C**) AISI Index. (**D**–**F**) are the directions and magnitude of the assigned weights for each log-transformed metal in relation to inflammation in female population in quantile g-computation. (**D**) HALP Index, (**E**) SIRI Index, (**F**) AISI Index. The model was adjusted for age, race, educational level, marital status, annual family income, alcohol status, smoking status, physical activities and BMI.

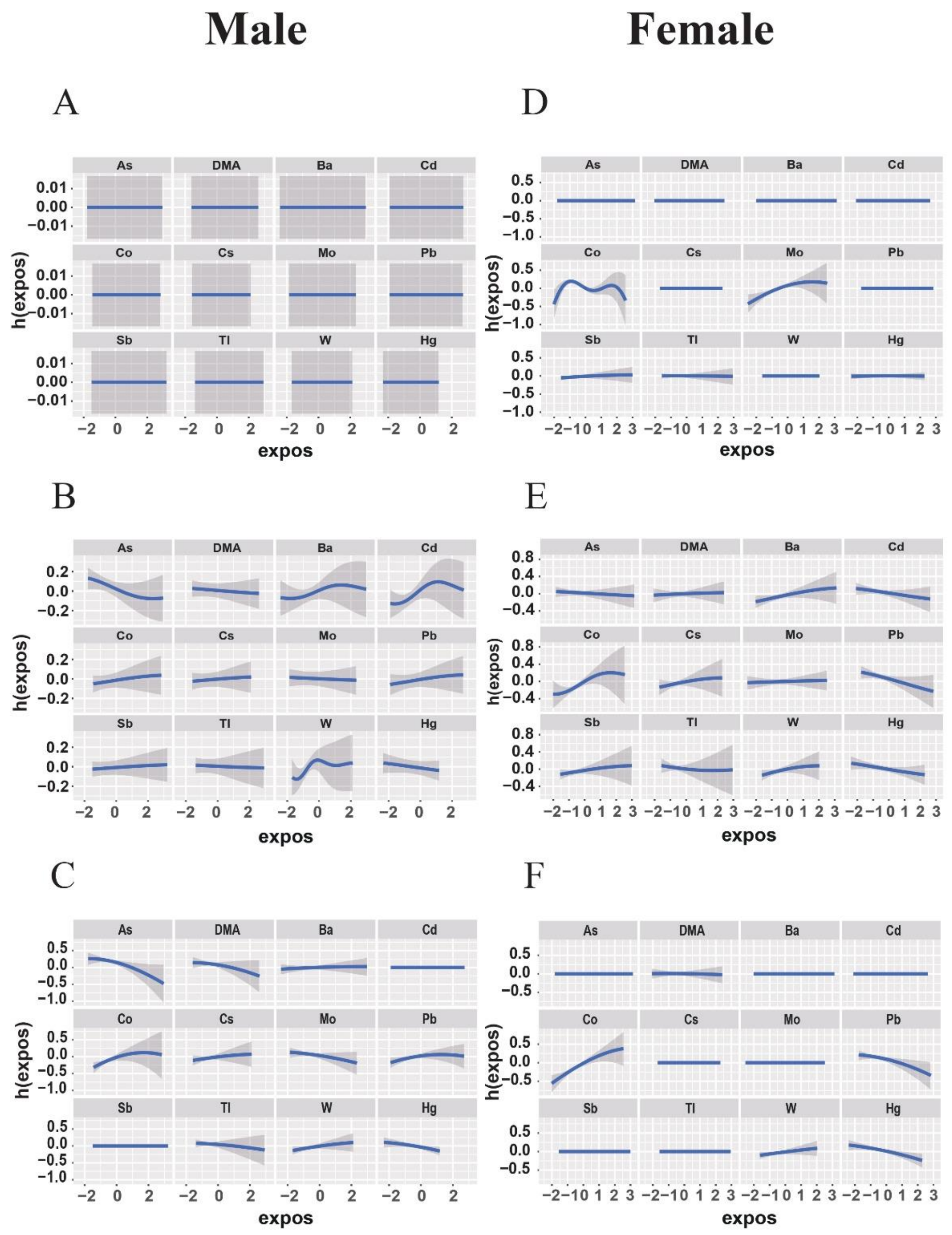

Figure S7. In the BKMR model, when the concentrations of all other metals were fixed at the median level, the exposure-response relationship function between a single metal and each inflammation index. (**A**–**C**) are the relationships in male population, (**A**) HALP Index, (**B**) SIRI Index, (**C**) AISI Index. (**D**–**F**) are the relationships in female population, (**D**) HALP Index, (**E**) SIRI Index, (**F**) AISI Index. The model was adjusted for age, race, educational level, marital status, annual family income, alcohol status, smoking status, physical activities and BMI.

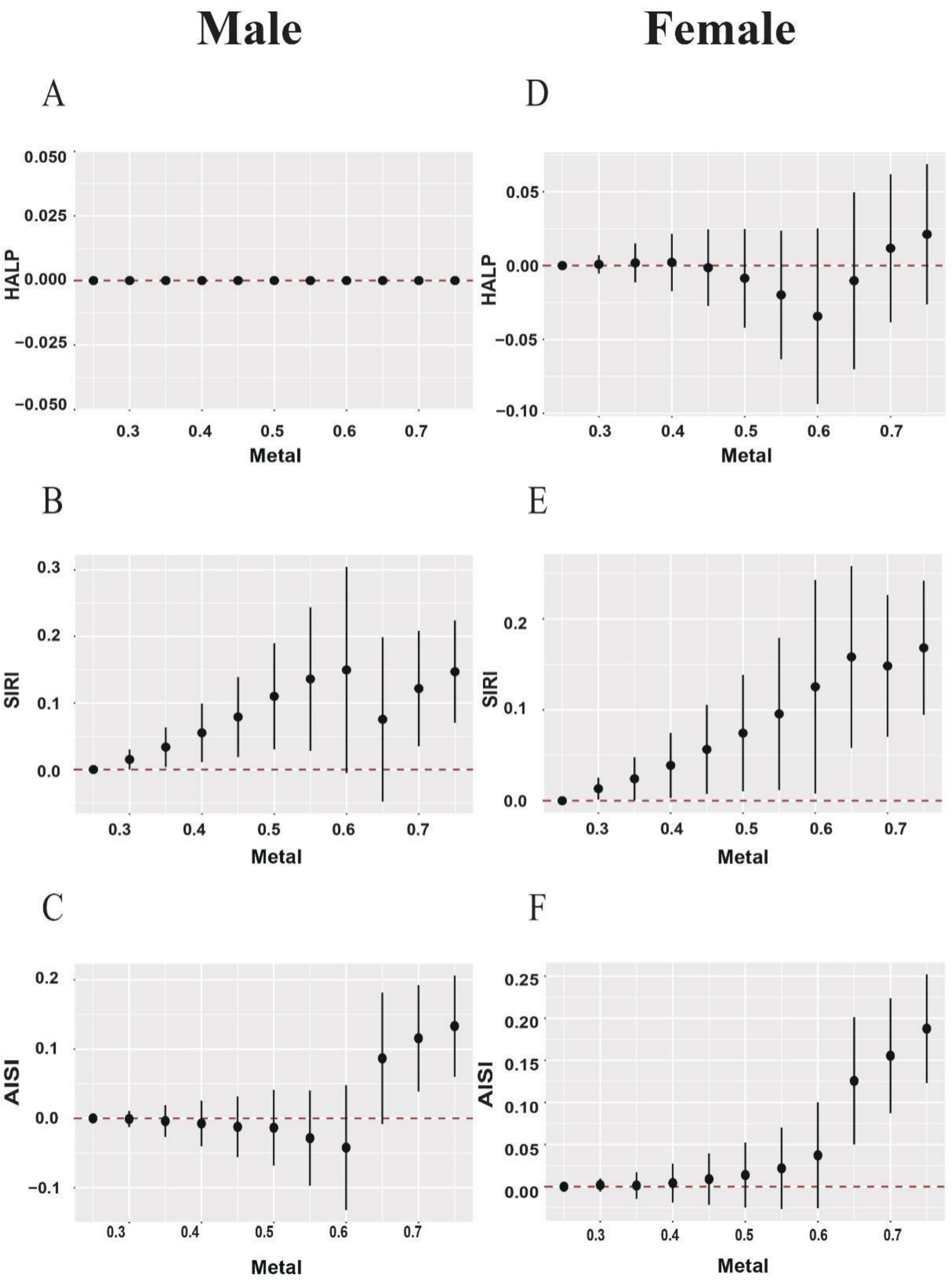

Figure S8. The joint association of mixed exposure of metal in the BKMR model. (**A**–**C**) are the relationships in male population, (**A**) HALP Index, (**B**) SIRI Index, (**C**) AISI Index. (**D**–**F**) are the relationships in female population, (**D**) HALP Index, (**E**) SIRI Index, (**F**) AISI Index. The model was adjusted for age, race, educational level, marital status, annual family income, alcohol status, smoking status, physical activities and BMI.

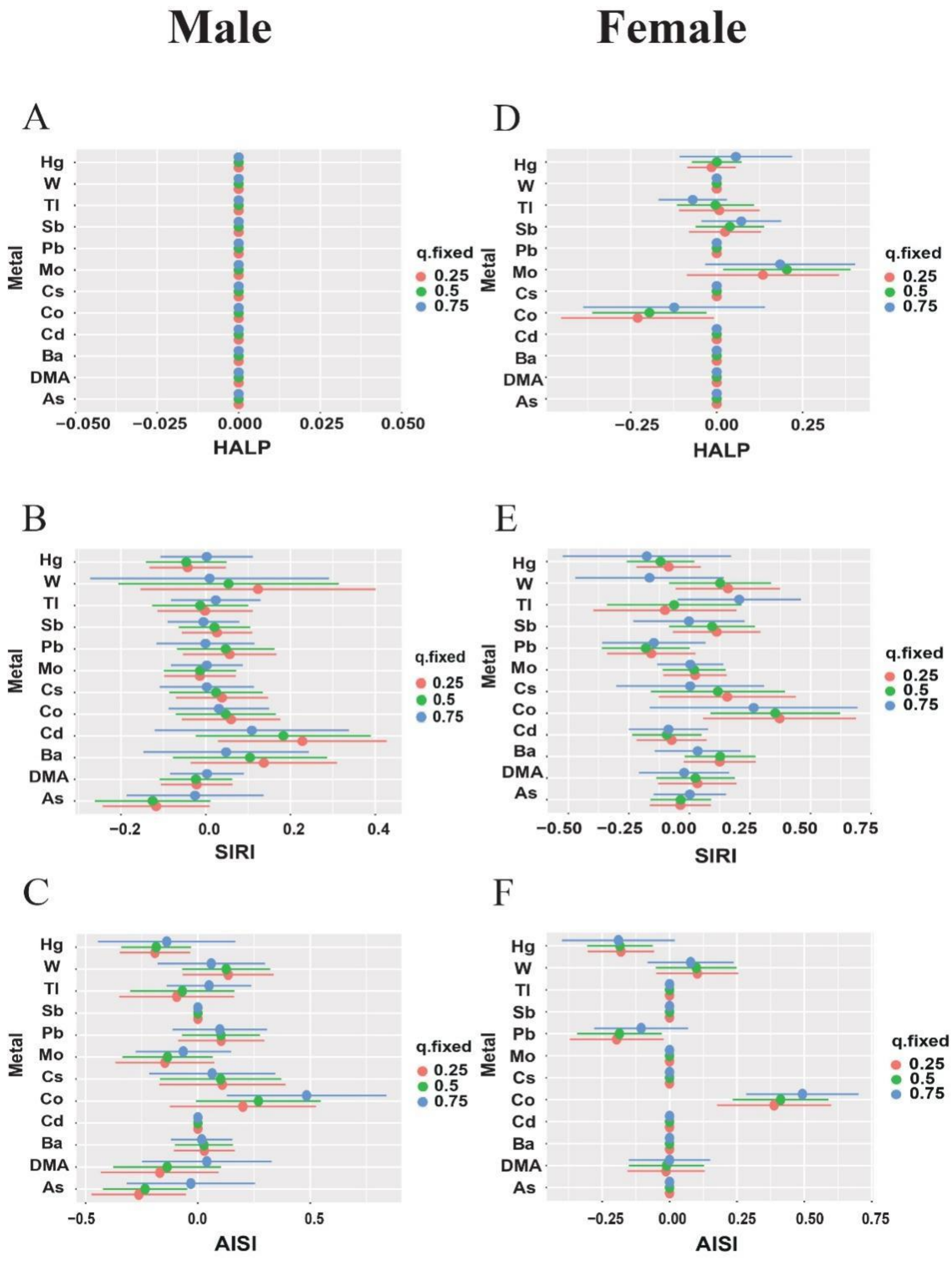

Figure S9. This figure describes the estimated difference in inflammation index for each metal from the 25th to the 75th percentile when all other metals are fixed at the 25th (red line), 50th (green line), or 75th percentile (blue line). The point represents the estimated value, and the horizontal line represents the 95% confidence interval (CI). (**A**–**C**) are the relationships in male population, (**A**) HALP Index, (**B**) SIRI Index, (**C**) AISI Index. (**D**–**F**) are the relationships in female population, (**D**) HALP Index, (**E**) SIRI Index, (**F**) AISI Index. The model was adjusted for age, race, educational level, marital status, annual family income, alcohol status, smoking status, physical activities and BMI.

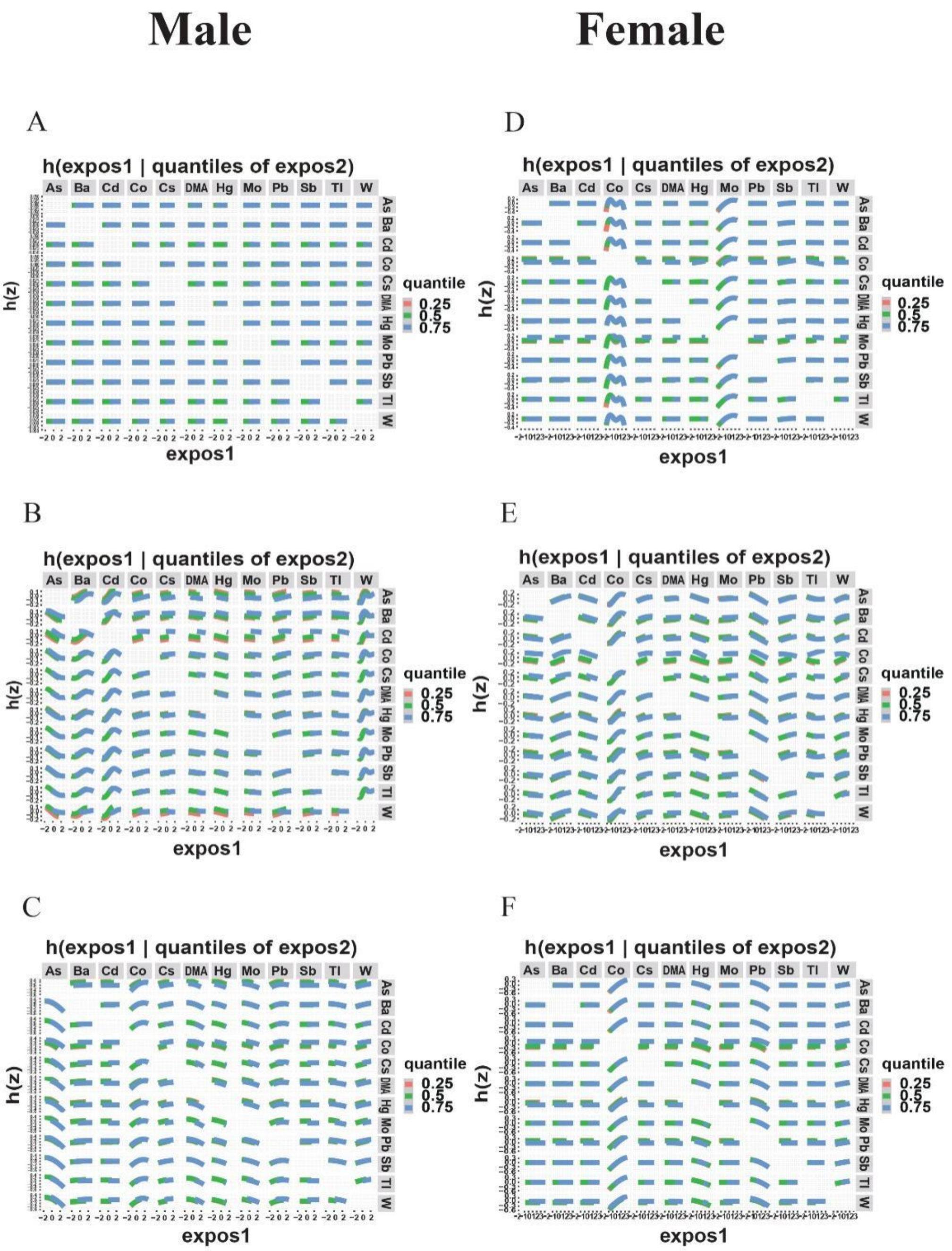

Figure S10. This figure is a bivariate exposure response function of metal exposure and inflammation index when a metal is fixed in different (25th , 50th ,75th) percentiles and other metals are fixed at the 50th percentile, the average difference between the other metal and the inflammation index as a bivariate exposure response function. (**A**–**C**) are the relationships in male population, (**A**) HALP Index, (**B**) SIRI Index, (**C**) AISI Index. (**D**–**F**) are the relationships in female population, (**D**) HALP Index, (**E**) SIRI Index, (**F**) AISI Index. The model was adjusted for age, race, educational level, marital status, annual family income, alcohol status, smoking status, physical activities and BMI.
